# Integrated analysis identifies AQP9 correlates with immune infiltration and acts as a prognosticator in multiple cancers

**DOI:** 10.1038/s41598-020-77657-z

**Published:** 2020-11-27

**Authors:** Xiaohong Liu, Qian Xu, Zijing Li, Bin Xiong

**Affiliations:** 1 Department of Gastrointestinal Surgery, Zhongnan Hospital of Wuhan University, Wuhan, 430071 People’s Republic of China; 2 Department of Gastric and Colorectal Surgical Oncology, Zhongnan Hospital of Wuhan University, Wuhan, 430071 People’s Republic of China; 3 Hubei Key Laboratory of Tumor Biological Behaviors, Wuhan, 430071 People’s Republic of China; 4 Hubei Cancer Clinical Study Center, Wuhan, 430071 People’s Republic of China

**Keywords:** Cancer, Computational biology and bioinformatics, Immunology, Biomarkers

## Abstract

Aquaporin 9 (AQP9), as an aquaglyceroporin, is expressed in many immune cells and plays important role in tumor initiation and progression. However, the relationship between AQP9 and tumor-infiltrating cells, and its prognostic value in cancers still require comprehensive understanding. Herein, we aimed to elucidate the correlations of AQP9 with prognosis and immune infiltration levels in diverse cancers. We detected the expression and survival data of AQP9 through Oncomine, TIMER, Kaplan–Meier Plotter and PrognoScan databases. The correlations between AQP9 and immune infiltrates were analyzed in TIMER database. Our results found that high AQP9 expression was significantly correlated with worse prognosis in breast, colon and lung cancers, while predicted better prognosis in gastric cancer. Moreover, AQP9 had significant association with various immune infiltrating cells including CD8^+^ and CD4^+^ T cells, neutrophils, macrophages and dendritic cells (DCs), and diverse immune gene markers in BRCA, COAD, LUAD, LUSC and STAD. AQP9 was also significantly correlated with the regulation of tumor associated macrophages (TAM). These results indicate that AQP9 can play as a significant biomarker to determine the prognosis and the immune infiltrating levels in different cancers. It might also contribute to the development of the immunotherapy in breast, colon, lung and gastric cancers.

## Introduction

Cancer has been a worldwide health problem with a high risk of mortality^1^. However, the prognosis of multiple cancers still remains unfavorable. Efficient treatment is vital for improving the prognosis of patients diagnosed with cancer. Immune-related mechanisms have been reported to participate in tumorigenesis and development of cancers^2^. Therefore, immunotherapeutic strategies have been regarded as a promising direction in the clinical treatment of tumors^3^. Studies have been reported that programmed cell death protein 1 (PD-1) is a co-inhibitory receptor expressed in a variety of immune cells and programmed cell death1 ligand 1 (PDL1) is associated with immune evasion of many tumor types^4^. Cytotoxic T lymphocyte associated antigen 4 (CTLA4) is a homologous gene for CD28, a key co-stimulatory receptor on T cells, interfering with immune synapses and T cell inactivation^5^. Lymphocyte activation gene 3 (LAG-3) is expressed in NK cells and activated T cells and blockade of LAG-3 promotes T-cell proliferation, activation and effector function^6^. Unfortunately, response rates to immunotherapy, such as anti-PD-1, anti-PD-L1, anti-CTLA4 and anti-LAG3 remain low and partial, and more efforts are needed to improve the outcome of these treatments^7–10^. The development of prognostic biomarkers or precise immunotherapeutic targets can help overcome these limitations^3^. In addition, the presence or absence of immune infiltrating cells may lead to changes in the tumor microenvironment^11^. Increasing researches have reported that tumor infiltrating leucocytes, such as tumor-infiltrating neutrophils (TIN) and tumor associated macrophages (TAM) have a profound impact on the efficacy of immunotherapy and clinical prognosis of patients^12^. Therefore, it is very necessary and urgent to clarify the immunophenotype of tumor-immune interaction and search for new immune-related therapeutic targets to improve the prognosis of cancer patients.

Aquaporin 9 (AQP9) is a member of the aquaporin (AQP) family that transports water alone or water with small solutes such as glycerol. In addition to water transport, AQP9 was also involved in the occurrence and development of various tumors, promoting the proliferation, migration and invasion of tumor cells^13^. Increasing studies have revealed the involvement of AQP9 in tumor progression: AQP9 has taken a part in the growth and migration of prostate cancer cells^14^. AQP9 can inhibit the invasion of liver cancer cells and the proliferation of xenograft tumors^15^. In brain tumors, the motility and invasion of astrocytoma cells can be suppressed by the down-regulation of AQP9, while the overexpression of AQP9 promotes the migration and invasion of astrocytoma cells^16^. Moreover, AQP9 can also activate RAS signal and sensitize tumor cells to chemotherapy drugs in colorectal cancer^17^. Therefore, AQP9 may be an important target related to a variety of cancers. However, only a few studies have been reported to elucidate the correlations between AQP9 and immune function. AQP9 is necessary for the inflammatory response and DCs maturation, and the expression level of AQP9 was significantly upregulated after being exposed to LPS^18^. AQP9-expressing neutrophils are also reported to be crucial to the establishment of contact hypersensitivity in mice^19^. Nevertheless, the underlying function and mechanisms of AQP9 in tumor progression and tumor immunology remain unelucidated. Therefore, it is necessary to focus on the correlation and potential mechanism between AQP9 and immune infiltration in different tumors.

In this study, we comprehensively performed an in-depth analysis on AQP9 expression and its impact on prognosis in different cancers, and we also probed the association of AQP9 expression with tumor immune cell infiltrating levels in breast invasive carcinoma (BRCA), colon adenocarcinoma (COAD), lung adenocarcinoma (LUAD), lung squamous cell carcinoma (LUSC) and stomach adenocarcinoma (STAD). We further explored the potential mechanisms that AQP9 might participate in the regulation of tumor infiltration. Our findings in the study may throw new light on the significant role of AQP9 in breast, colon, lung and gastric cancers, as well as provide a new direction for further study of potential mechanisms between AQP9 and tumor-immune interactions.

## Results

### The expression of AQP9 in different human cancers

**Figure 1:**
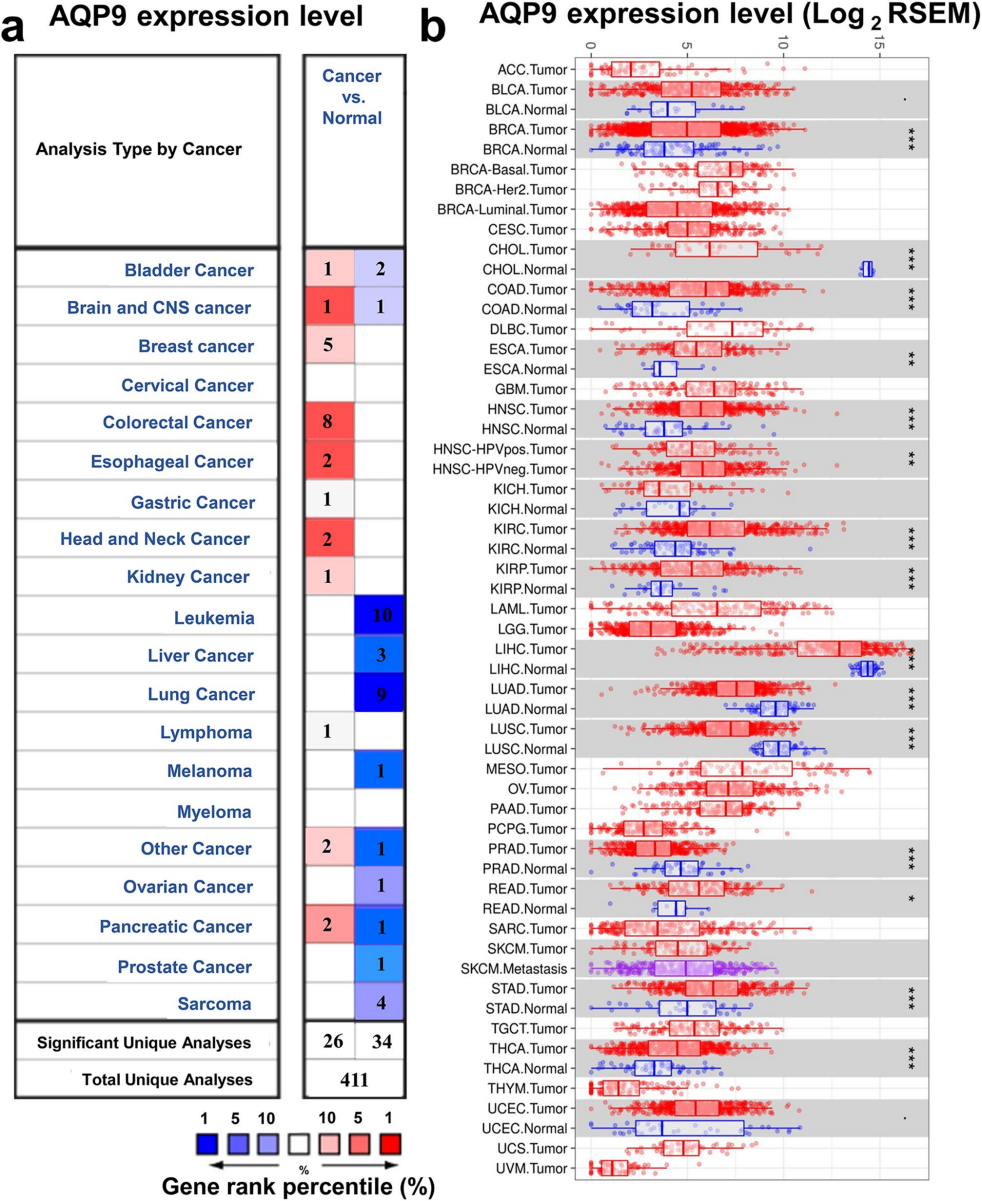
Expression of AQP9 in different human tumors. (**a**) Differently expressed AQP9 in various cancers in the Oncomine database. (**b**) AQP9 expression levels in diverse tumor and normal tissues were analyzed in TIMER database (*P < 0.05, **P < 0.01, ***P < 0.001).

To evaluate the discrepancies of AQP9 mRNA expression levels in different tumor and normal tissues, we analyzed the Oncomine database to determine the AQP9 expression in multiple cancers. The results demonstrated that the AQP9 expression was up-regulated in breast, colorectal and gastric cancers (Fig. 1a), while decreased in lung cancer compared with their normal tissues. The detailed information of AQP9 expression in different cancers were summarized in Supplementary Table S1, which suggested AQP9 mRNA expression levels have significant differences in multiple malignances. Furthermore, we evaluated the protein expression levels of AQP9 via HPA database, and the result revealed that the protein expression level of AQP9 was low in BRCA and LUSC, while the protein level of AQP9 in COAD, LUAD and STAD were not detected (Supplementary Fig. S1).

In Fig. 1b, we analyzed the RNA-seq data of different cancers to further evaluate the differences of AQP9 expression. The AQP9 expression level was significantly up-regulated in BRCA, COAD, STAD compared with normal tissues. Nevertheless, the AQP9 expression was significantly reduced in LUAD and LUSC than in adjacent normal tissues. The results corresponded with the previous microarray analysis.

In order to investigate the potential factors that may affect the AQP9 expression, we explored the association of AQP9 expression levels with relevant gene mutation levels in different cancers via LinkedOmincs database (Supplementary Fig. S2) and the promoter methylation levels of AQP9 in UALCAN database (Supplementary Fig. S3). It showed that higher AQP9 expression was associated with the mutant of TP53 in BRCA and BRAF in COAD. The promoter methylation levels of AQP9 were significantly reduced in COAD and LUSC, compared with that in the normal tissues. These results suggested that the mutation of some tumor related genes and promoter methylation level might serve a potential part in the expression of AQP9 in some specific cancer types.

### Prognostic potential of AQP9 in cancers

**Figure 2:**
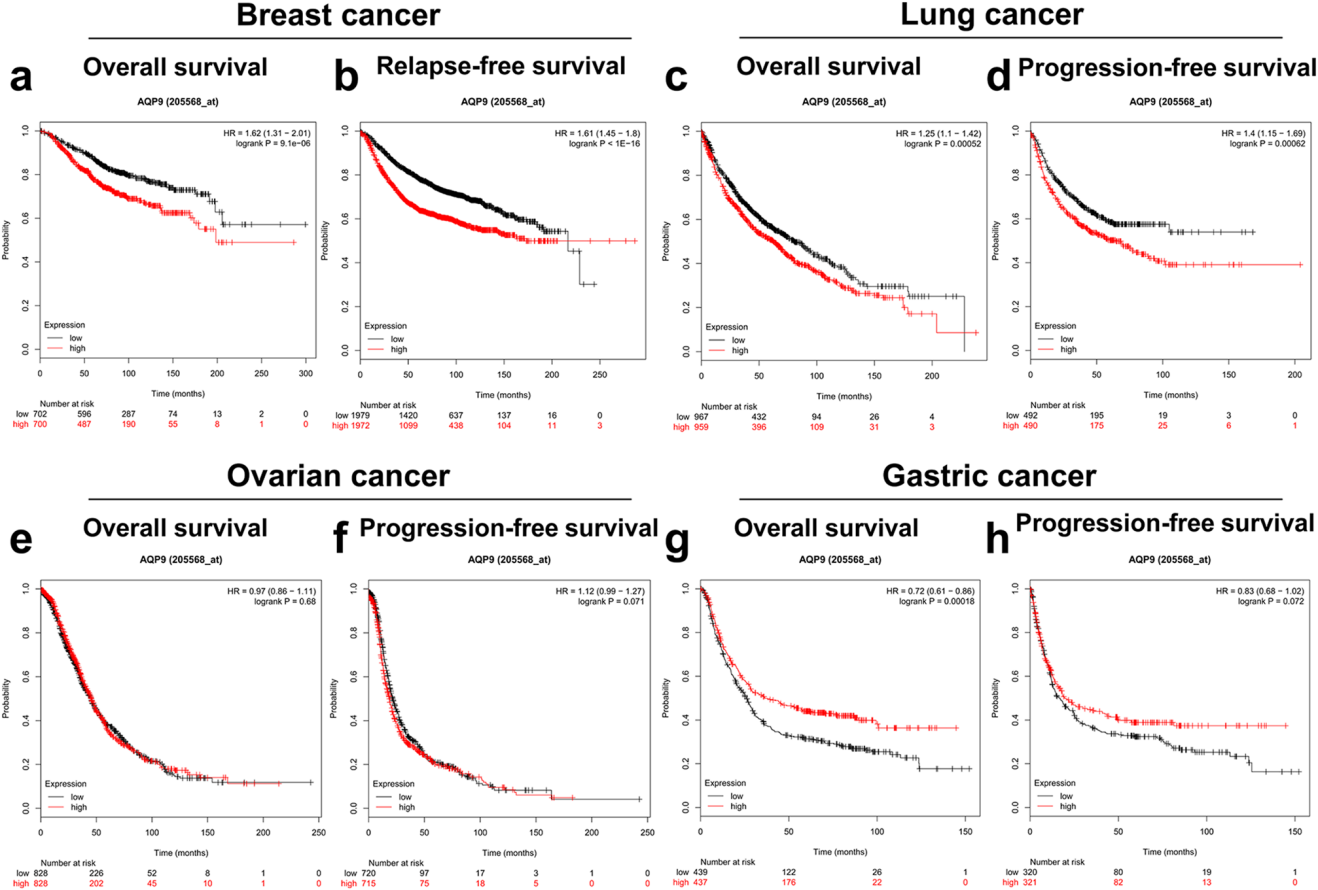
Comparison of survival curves of the high and low AQP9 expression in different cancers in the Kaplan–Meier plotter. High expression of AQP9 was correlated with poor prognosis of OS and RFS in (**a**,**b**) breast cancer (n = 993, n = 2519) and (**c**,**d**) lung cancer (n = 828, n = 982). (**e**,**f**) AQP9 expression had no significant correlation with the prognosis of ovarian cancer (n = 428, n = 1435). (**g**,**h**) High AQP9 expression predicted favorable prognosis of OS and PFS in gastric cancer (n = 876, n = 641). *OS* overall survival, *RFS* relapse-free survival, *PFS* progression-free survival.

Prognostic analysis is a key point of tumor related research in recent years. Subsequently, we analyzed the prognostic significance of AQP9 in diverse tumors in Kaplan–Meier plotter database (Fig. 2a–h). The result revealed that the higher AQP9 expression was determined to associate with worse prognosis in patients with breast or lung cancers. However, overexpression of AQP9 was observed to have better impact on the prognosis in gastric cancer. Nevertheless, AQP9 expression showed less influence on the prognosis of ovarian cancer. In conclusion, the expression of AQP9 can make a profound difference to the prognosis of breast, lung and gastric cancers.

We also analyzed the prognostic significance of AQP9 in patients stratified with different clinical characteristics in breast, gastric and lung cancers through the Kaplan–Meier plotter (Supplementary Tables S2–S4). It was found that the overexpression of AQP9 was correlated with the prognosis at different lymph node status in BRCA, among which high AQP9 expression predicted poorer OS and RFS prognosis in Lymph node negative BRCA, and poorer RFS prognosis in Lymph node positive BRCA (Supplementary Table S2). However, overexpression of AQP9 in gastric cancer was related to better prognosis of patients at stage1 and stage3. The high AQP9 expression was also found to have better impact on the prognosis of patients at stage N_0_, N_2_ and N_1+2+3_ in gastric cancer. Moreover, the overexpression of AQP9 was also correlated with better prognosis of patients without distant metastasis in gastric cancer, while AQP9 expression had no significant association with the prognosis of patients with distant metastasis (Supplementary Table S3). In addition, the AQP9 expression in lung cancer had poorer impact on the prognosis of patients at stage N_0_ and stage N_1_ (Supplementary Table S4). The results suggested that high AQP9 expression correlated with the prognosis of patients with lymphatic metastasis in breast, gastric and lung cancers.

**Figure 3:**
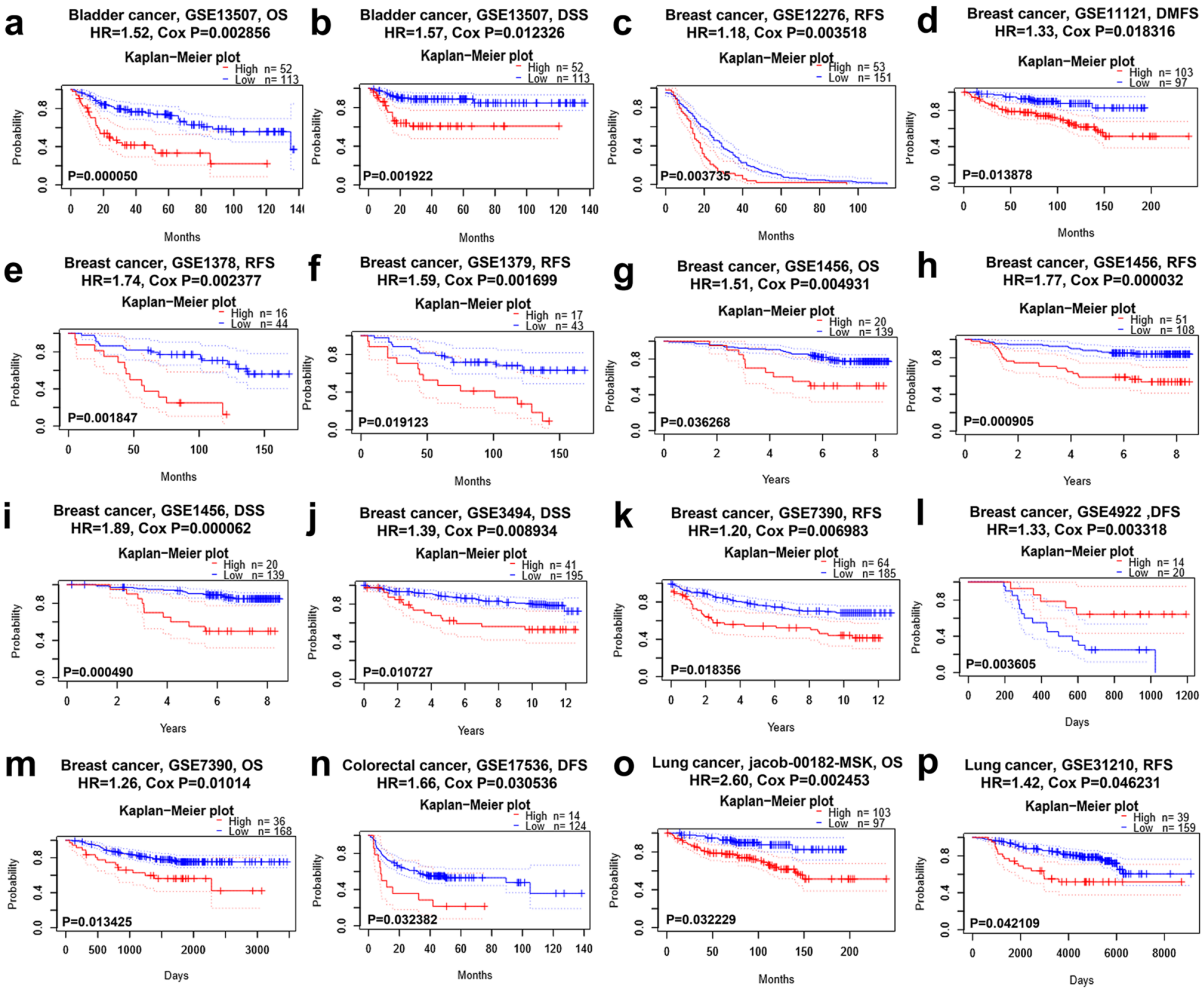
Survival analysis of AQP9 expression in various cancer types from PrognoScan database. (**a**,**b**) OS and DSS survival curves in bladder cancer cohort (GSE13507, n = 165). (**c**–**m**) High AQP9 expression had poor OS, RFS, DFS, DMFS in eight breast cancer cohorts [GSE1456 (n = 159), GSE1379 (n = 60), GSE1378 (n = 60), GSE4922 (n = 249), GSE12276 (n = 204), GSE7390 (n = 249), GSE3494 (n = 236), GSE11121 (n = 200)]. (**n**) High expression of AQP9 predicted poorer DFS in colorectal cancer than low AQP9 expression (GSE17536, n = 145). (**o**,**p**) High AQP9 expression correlated with poor OS, RFS in two lung cancer cohorts [jacob-00182-MSK (n = 104), GSE31210 (n = 204)]. *OS* overall survival, *DFS* disease-free survival, *RFS* relapse-free survival, *DSS* disease-specific survival, *DMFS* distant metastasis-free survival.

We further examined the potential impact of the AQP9 expression on survival rate in multiple cancers via PrognoScan database (Fig. 3 and Supplementary Fig. S4). We found that the AQP9 expression level had significant impact on the prognosis in four types of cancers involving bladder, breast, colorectal and lung cancers. Eight cohorts of breast cancer (GSE1456, GSE1379, GSE1378, GSE4922, GSE12276, GSE7390, GSE3494, GSE11121) revealed that high expression level of AQP9 correlated with poor prognosis (Fig. 3c–m). Furthermore, one cohort (GSE17536) revealed that high AQP9 expression predicted worse prognosis for colorectal cancer patients (Fig. 3n). And two cohorts (jacob-00182-MSK, GSE31210) of lung cancer revealed that overexpression of AQP9 had poorer impact on the prognosis (Fig. 3o–p). The result suggested that AQP9 may play as an important biomarker that predicts poor prognosis for breast, colorectal and lung cancers and favorable prognosis for gastric cancer.

### AQP9 expression is correlated with immune infiltration levels in breast, colon, lung and gastric cancers

**Figure 4:**
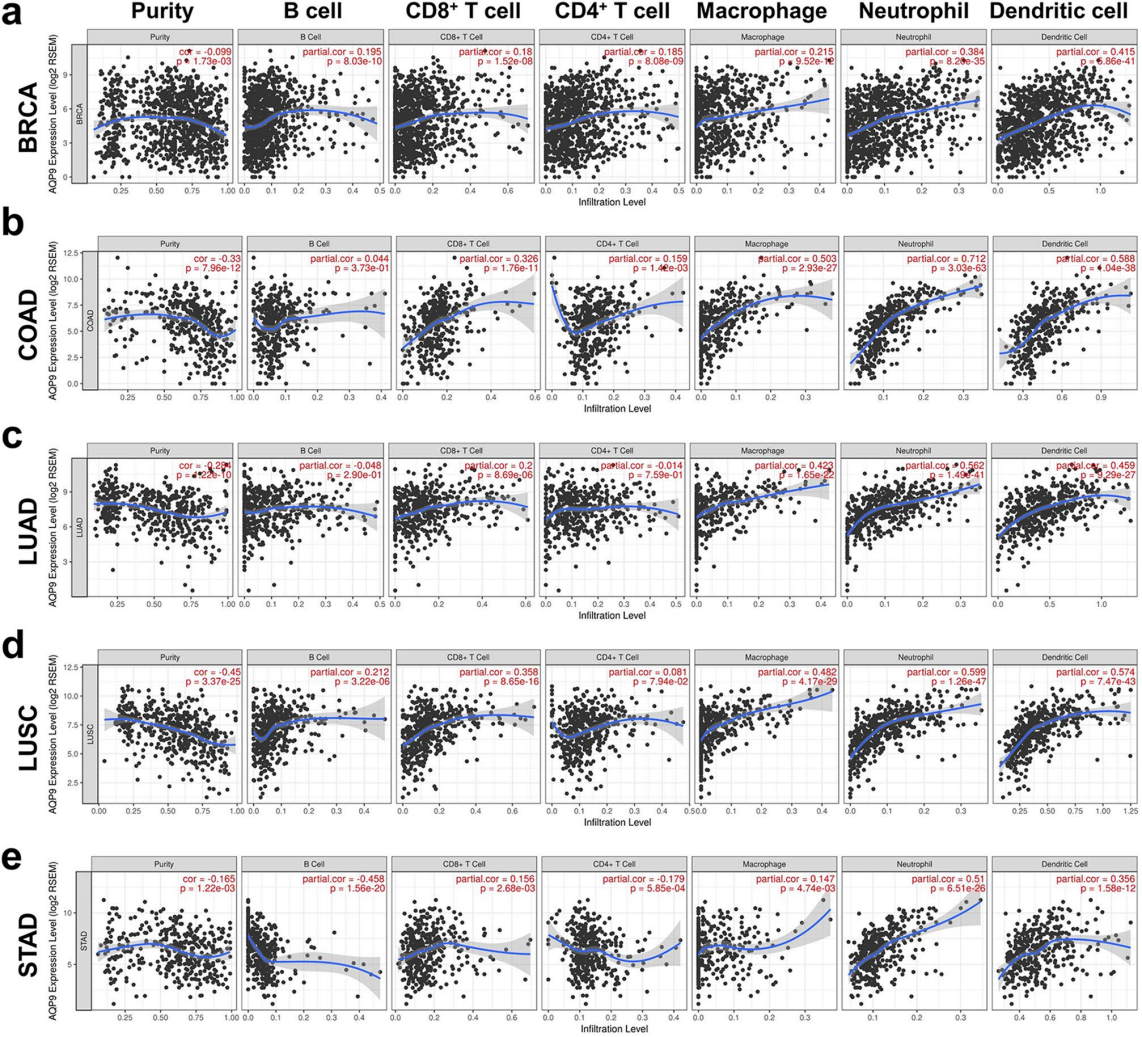
AQP9 expression correlated with immune infiltrating cells in BRCA, COAD, LUAD, LUSC and STAD. (**a**) AQP9 expression was positively correlated with infiltrating immune cells in BRCA (n = 1093). (**b**) Immune infiltration levels, except B cells, are positively related to AQP9 expression in COAD (n = 457). (**c**) AQP9 expression has positive correlations with infiltrating levels, except B and CD4^+^ T cells, in LUAD (n = 515). (**d**) Infiltrating levels, other than CD4^+^ T cells, in LUSC have positive correlations with AQP9 expression (n = 501). (**e**) AQP9 expression is negatively correlated with B cells and CD4^+^ T cells infiltrating levels, while positively with that of CD8^+^ T cells, macrophages, neutrophils, and dendritic cells in STAD (n = 415).

Immune-infiltrating cells in tumor tissues can not only perturb the cytokine signal in tumor microenvironment but also serve a significant part in cancer biology^20^. Tumor infiltrating lymphocytes are important predictors for the status of sentinel lymph node and prognosis of cancer patients^21^. In our analysis, we explored 39 types of cancer in TIMER to determine whether AQP9 expression was related to the abundance of immune infiltration in diverse cancers (Fig. 4 and Supplementary Fig. S5).

In view of the correlation of AQP9 expression with immune infiltrating levels in diverse cancers, we then examined the specific cancer types in which AQP9 can play a role as a prognostic biomarker and is significantly associated with immune infiltration levels. We chose distinct cancer types whose prognosis correlated significantly with AQP9 expression in Prognoscan and Kaplan–Meier plotter databases. Considering that the tumor purity is a key factor influencing the genomic analysis of immune infiltrates^22^, we further screened out cancer types that had negative correlations with tumor purity. Eventually, it was found that AQP9 expression level was significantly correlated with both clinical prognosis and tumor-infiltrating immune cells in BRCA, COAD, LUAD, LUSC and STAD. Our findings showed that there was a significant positive correlation between AQP9 expression and immune infiltrates, such as CD8^+^T cells, neutrophils, macrophages and DCs in BRCA (Fig. 4a), COAD (Fig. 4b), LUAD (Fig. 4c), LUSC (Fig. 4d) and STAD (Fig. 4e). For the B cells, AQP9 expression showed a positive correlation with them in BRCA and LUSC, while a moderately negative correlation in STAD. For CD4^+^T cells, there was a significantly positive association between them and AQP9 expression in both BRCA and COAD, while a negative association in STAD. These results strongly indicated that AQP9 might have an important effect on tumor immune infiltration, especially on the infiltrating levels of neutrophils, macrophages and DCs in breast, colorectal, lung and gastric cancers.

### Correlations of AQP9 expression with immune marker sets

**Figure 5:**
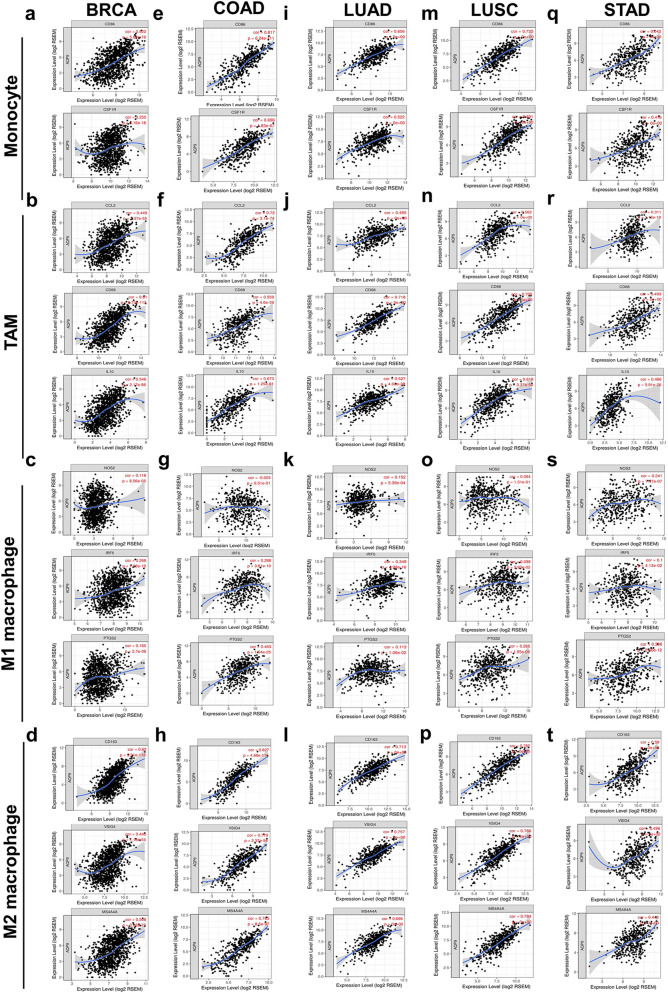
AQP9 expression was correlated with the polarization of macrophage in BRCA, COAD, LUAD, LUSC and STAD. Correlations of AQP9 expression with gene markers for monocytes, TAMs, M1 and M2 macrophages in (**a**–**d**) BRCA, (**e**–**h**) COAD, (**i**–**l**) LUAD, (**m**–**p**) LUSC and (**q**–**t**) STAD.

To further explore the association of AQP9 expression with various immune-infiltrating cells, we intensively conducted the correlation analysis between AQP9 and the representative marker genes of different immune infiltrating cells through TIMER and GEPIA databases (Fig. 5 and Supplementary Tables S5, S6). Different functional T cells, including several T-helper cells, Tregs and exhausted T cells were also investigated. The correlation of AQP9 with diverse gene markers were adjusted by purity. The results showed that AQP9 significantly correlated with most of the immune markers in BRCA, COAD, LUAD, LUSC and STAD.

Actually, we found the majority of gene markers for monocytes, TAMs, M1 and M2 macrophages were significantly correlated with AQP9 expression in BRCA, COAD, LUAD, LUSC and STAD (Fig. 5 and Supplementary Table S6). To be more specific, the results revealed that AQP9 expression were significantly correlated with gene markers, such as CD86 and CD115 for monocytes, CD68, IL10, CCL-2 for TAMs, IRF5, PTGS2 for M1 macrophages, CD163, MS4A4A and VSIG4 of M2 macrophages in breast, colon, lung and gastric cancers (P < 0.05; Fig. 5a–t). The correlation result of AQP9 expression with above markers was also evaluated via GEPIA, which was consistent with those in the TIMER database (Supplementary Table S6). The results suggested that AQP9 might correlate with macrophage polarization in BRCA, COAD, LUAD, LUSC and STAD.

The AQP9 expression was also observed to have significant correlation with the marker sets of neutrophils, DCs, Treg cells and T cell exhaustion (Supplementary Table S5). Specifically, CD11b (ITGAM) of neutrophils was moderately to strongly associated with AQP9 expression in COAD, LUAD, LUSC and STAD, while weakly correlated with the AQP9 expression in BRCA. AQP9 expression was also significantly correlated with the expression of DC markers such as HLA-DPB1, HLA-DRA and CD11c. In addition, there was a significantly positive correlation of AQP9 expression with the gene markers of Treg cells including FOXP3, CCR8 and TGFB1 in BRCA, COAD, LUAD, LUSC and STAD. Moreover, AQP9 expression had a significant correlation with and the marker sets of exhausted T cells including PD-1, CTLA4, LAG3, TIM-3 as well as GZMB. Furthermore, TIM-3 acted as an essential part in the regulation of T cell exhaustion and was strongly associated with AQP9 expression, which suggested that increased AQP9 expression served a significant part in T cell exhaustion mediated by TIM-3. As a result, the findings further confirmed that AQP9 expression was closely correlated with immune infiltrates in BRCA, COAD, LUAD, LUSC and STAD, which indicated that AQP9 was crucial in immunologic escape and immune tolerance in tumor microenvironment.

### GO (gene ontology) analysis of genes co-expressed with AQP9 in BRCA, COAD, LUAD, LUSC and STAD

To investigate the potential mechanism of AQP9 affecting tumor infiltrating levels, genes significantly correlated with AQP9 in BRCA, COAD, LUAD, LUSC and STAD were analyzed in LinkedOmics database to illuminate the possible GO sets that might be involved in the regulation of tumor-immune interaction mechanisms. The detailed results of the GO analysis demonstrated that with respect to biological processes (BP), genes co-expressed with AQP9 were significantly enriched in immune modules, such as ‘lymphocyte mediated immunity’. With respect to cellular component (CC), genes co-expressed with AQP9 were significantly enriched in ‘MHC protein complex’, and with respect to molecular function (MF), genes co-expressed with AQP9 were significantly enriched in ‘MHC protein binding’ and ‘immunoglobulin binding’ in BRCA, COAD, LUSC and STAD (Table 1).

**Table 1:** The gene ontology analysis of genes co-expressed with AQP9 in LinkedOmics database.

Cancer	GO Term	GeneSet	Description	Count	p-value	FDR
BRCA	BP	GO:0002250	Adaptive immune response	368	0	0
		GO:0045088	Regulation of innate immune response	350	0	0
		GO:0036230	Granulocyte activation	476	0	0
		GO:0002446	Neutrophil mediated immunity	473	0	0
		GO:0002694	Regulation of leukocyte activation	461	0	0
		GO:0002764	Immune response-regulating signaling pathway	452	0	0
		GO:0032615	Interleukin-12 production	53	0	0
		GO:0032612	Interleukin-1 production	86	0	0
		GO:0002449	Lymphocyte mediated immunity	229	0	0
		GO:0042110	T cell activation	439	0	0
		GO:0002683	Negative regulation of immune system process	392	0	0
		GO:0050727	Regulation of inflammatory response	349	0	0
		GO:0002697	Regulation of immune effector process	365	0	0
		GO:0032635	Interleukin-6 production	120	0	0
	CC	GO:0042611	MHC protein complex	19	0.006309	0.005403
		GO:0001772	Immunological synapse	32	0.002899	0.007356
	MF	GO:0042287	MHC protein binding	24	0	0.003098
		GO:0019865	Immunoglobulin binding	22	0	0.003835
COAD	BP	GO:0002269	Leukocyte activation involved in inflammatory response	31	0	0.007515
		GO:0032613	Interleukin-10 production	46	0	0.010735
		GO:0032633	Interleukin-4 production	34	0	0.011836
		GO:0042116	Macrophage activation	78	0	0.01362
		GO:0032612	Interleukin-1 production	86	0	0.01409
		GO:0071887	Leukocyte apoptotic process	103	0	0.014403
		GO:0032615	Interleukin-12 production	53	0	0.014703
		GO:0150076	Neuroinflammatory response	48	0	0.014859
		GO:0071706	Tumor necrosis factor superfamily cytokine production	133	0	0.014925
	CC	GO:0042611	MHC protein complex	19	0.008584	0.019271
	MF	GO:0019865	Immunoglobulin binding	22	0	0
		GO:0042287	MHC protein binding	24	0.006263	0.04006
LUAD	BP	GO:0045088	Regulation of innate immune response	125	0	0
		GO:0042110	T cell activation	149	0	0
		GO:0002694	Regulation of leukocyte activation	167	0	0
		GO:0070661	Leukocyte proliferation	98	0	0
		GO:0002250	Adaptive immune response	121	0	0.000551
		GO:0002697	Regulation of immune effector process	129	0	0.00058
		GO:0030101	Natural killer cell activation	24	0	0.000612
		GO:0032635	Interleukin-6 production	43	0	0.000648
		GO:0042116	Macrophage activation	28	0	0.000735
		GO:0002446	Neutrophil mediated immunity	147	0	0.000735
		GO:0002764	Immune response-regulating signaling pathway	159	0	0.000787
		GO:0002285	Lymphocyte activation involved in immune response	53	0	0.000826
		GO:0002449	Lymphocyte mediated immunity	85	0	0.000881
		GO:0002440	Production of molecular mediator of immune response	65	0	0.001502
		GO:0042113	B cell activation	66	0	0.002204
LUSC	BP	GO:0042116	Macrophage activation	78	0	0
		GO:0032635	Interleukin-6 production	120	0	0
		GO:0032612	Interleukin-1 production	86	0	0
		GO:0002446	Neutrophil mediated immunity	472	0	0
		GO:0050900	Leukocyte migration	394	0	0
		GO:0002250	Adaptive immune response	363	0	0
		GO:0032637	Interleukin-8 production	67	0	0
		GO:0002764	Immune response-regulating signaling pathway	452	0	0
	CC	GO:0042611	MHC protein complex	19	0	0.000887
		GO:0001772	Immunological synapse	32	0	0.001893
	MF	GO:0019865	Immunoglobulin binding	22	0	0
		GO:0042287	MHC protein binding	24	0.002283	0.005568
STAD	BP	GO:0002446	Neutrophil mediated immunity	473	0	0
		GO:0002764	Immune response-regulating signaling pathway	452	0	0
		GO:0032612	Interleukin-1 production	86	0	0
		GO:0002250	Adaptive immune response	368	0	0
		GO:0032623	Interleukin-2 production	63	0	0
		GO:0031349	Positive regulation of defense response	407	0	0
		GO:0032635	Interleukin-6 production	120	0	0
		GO:0006959	Humoral immune response	223	0	0
		GO:0002697	Regulation of immune effector process	365	0	0
		GO:0007159	Leukocyte cell–cell adhesion	310	0	0
		GO:0002694	Regulation of leukocyte activation	461	0	0
		GO:0002449	Lymphocyte mediated immunity	229	0	0
		GO:0002285	Lymphocyte activation involved in immune response	167	0	0
	CC	GO:0042611	MHC protein complex	19	0.009772	0.008677
		GO:0001772	Immunological synapse	32	0.003155	0.0107
	MF	GO:0019865	Immunoglobulin binding	22	0	0
		GO:0042287	MHC protein binding	24	0.006079	0.008962

Significant GO terms correlated with immunology were demonstrated in the table.

*BRCA* breast invasive carcinoma, *COAD* colon adenocarcinoma, *LUAD* lung Adenocarcinoma, *LUSC* lung squamous cell carcinoma, *STAD* stomach adenocarcinoma, *GO* gene ontology, *BP* biological process, *CC* cellular component, *MF* molecular function.

### KEGG (Kyoto encyclopedia of genes and genomes) pathway analysis of genes co-expressed with AQP9 in BRCA, COAD, LUAD, LUSC and STAD

**Figure 6:**
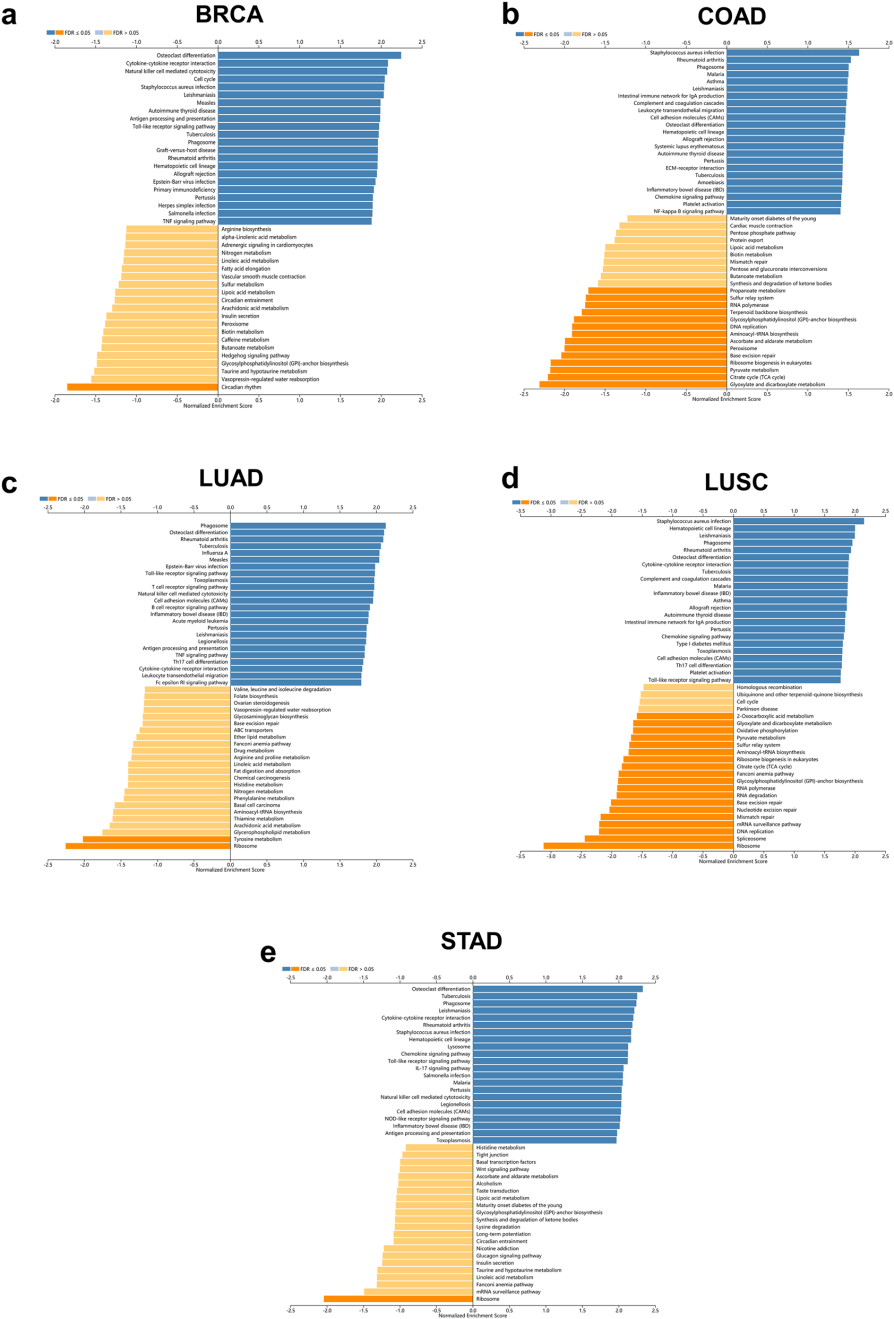
KEGG analysis of genes differentially expressed in correlation with AQP9. (**a**) Breast invasive carcinoma (BRCA), (**b**) colon adenocarcinoma (COAD), (**c**) lung adenocarcinoma (LUAD), (**d**) lung squamous cell carcinoma (LUSC) and (**e**) stomach adenocarcinoma (STAD) in the LinkedOmics database. Dark blue and dark yellow represent KEGG pathways in which the normal enrichment score (NES) is positive or negative and with a significance of FDR < 0.05. Light blue and light yellow represent KEGG pathways with a significance of FDR > 0.05.

KEGG pathway analysis was also obtained by LinkedOmics database to explore the potential mechanisms involved in tumor immunity. The results of the KEGG analysis (Fig. 6) illustrated the most significant pathways of genes co-expressed with AQP9 in breast, colon, lung and gastric cancers. The genes correlated with AQP9 were significantly enriched in pathways like ‘Natural killer cell mediated cytotoxicity’ in BRCA; ‘Leukocyte transendothelial migration’ in COAD; ‘T cell receptor signaling pathway’ in LUAD; ‘Toll-like receptor signaling pathway’ in LUSC; and ‘Chemokine signaling pathway’ in STAD. The genes co-expressed with AQP9 were found collectively enriched in ‘Toll-like receptor signaling pathway’ and ‘IL-17 signaling pathway’.

### Protein–protein interaction network of AQP9 and correlated immune genes in BRCA, COAD, LUAD, LUSC and STAD

**Figure 7:**
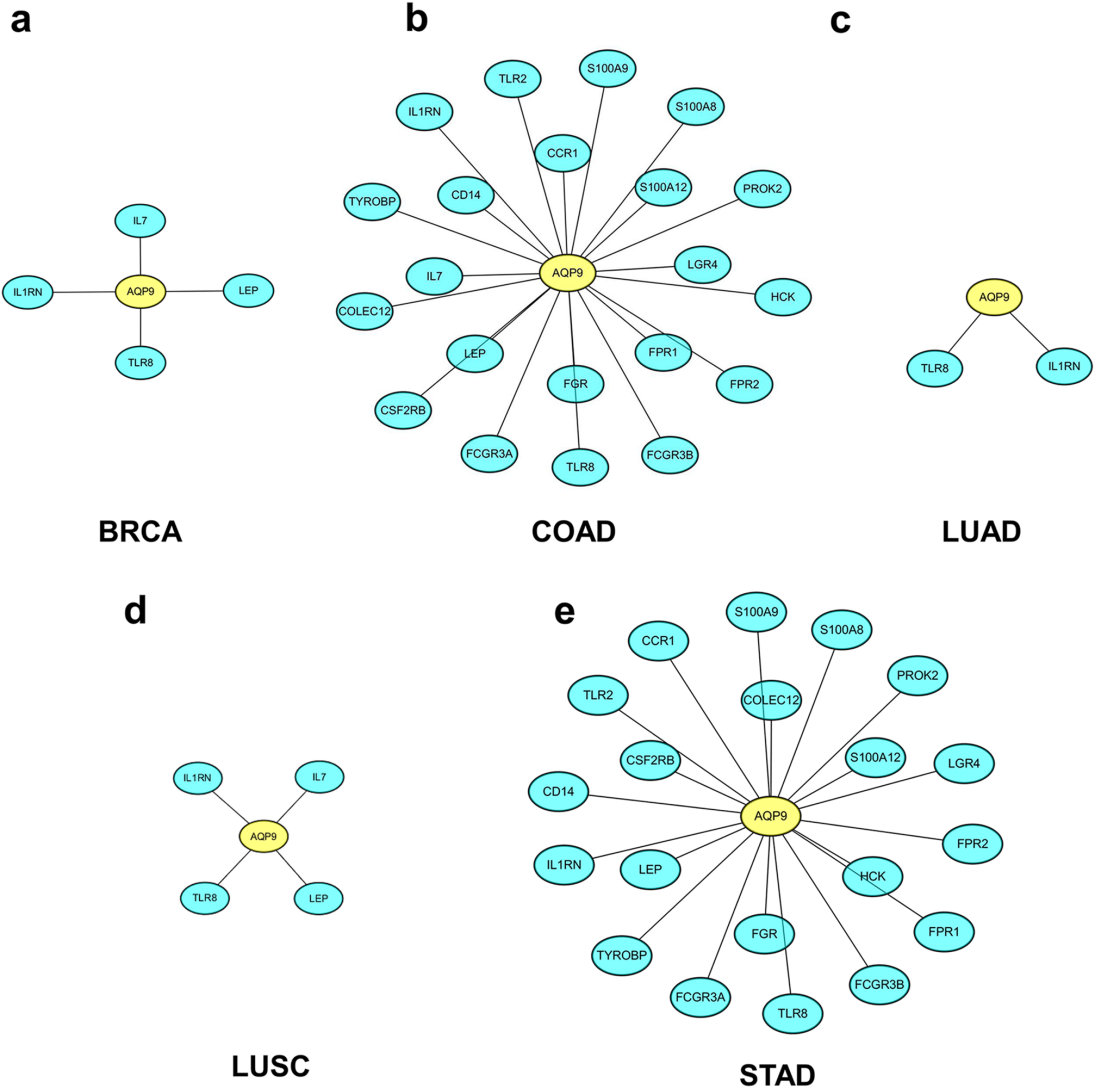
The protein–protein interaction network constructed for immune genes in correlation with AQP9. (**a**) BRCA (breast invasive carcinoma), (**b**) COAD (colon adenocarcinoma), (**c**) LUAD (lung adenocarcinoma), (**d**) LUSC (lung squamous cell carcinoma) and (**e**) STAD (stomach adenocarcinoma). Yellow and cyan represent AQP9 and the co-expressed genes, respectively.

In Fig. 7, we analyzed the interaction between AQP9 and the correlated immune genes in STRING database and Cytoscape. The immune related genes were download from the IMMPORT database (http://www.immport.org/) and then took the intersection with the previous obtained genes that were significantly correlated with AQP9. The results revealed that immune genes, such as TLR8 (Toll like receptor 8), IL-7 (Interleukin-7) and so on had a significant correlation with AQP9 in BRCA, COAD, LUAD, LUSC and STAD.

## Discussion

AQP9 is a water/glycerol channel protein with a broad substrate spectrum and is reported to serve as a significant part in the tumorigenesis and tumor progression^13,15^. Accumulating research elucidated a correlation between tumor-infiltrating cells and prognosis in various malignancies^2^. Immunotherapy has become a promising treatment method for advanced or metastatic cancers. ^3^ Therefore, there is an urgent need to develop more effective and accurate targets for immunotherapy. As it has been reported in previous studies, AQP9 was required for efficient DCs maturation and affected macrophage function by regulating cell volume, shape and protrusion development via water fluxes^18^. However, the potential mechanisms of AQP9 affecting tumor immune infiltration remained unclear. To gain more detailed insights into the underlying functions of AQP9 in tumor immune as well as its regulatory mechanism, we conducted bioinformatics analysis to guide further research as well as provide a biomarker for prognosis and clinical treatment in multiple cancers.

In this study, we provide an insight in the expression level of AQP9 as well as the potential regulating factors in correlation with AQP9 expression in diverse malignances. AQP9 mRNA expression level showed a significant difference in various cancer types, while AQP9 showed little difference in protein expression level. This result might indicate that the expression of AQP9 is affected by various regulatory mechanisms such as post-transcriptional level, translation level and post-translational level regulation. We also found that the mutant TP53 in BRCA and mutant BRAF in COAD were correlated with higher AQP9 expression. It has been reported that TP53 mutations have different clinical relevance in molecular subtypes of BRCA and TP53 can affect tumor energy metabolism such as increasing glycolysis^23^. Although TP53 is recognized as a tumor suppressor gene in various cancers, the mutant TP53 does not necessarily represent its inactivation as a tumor suppressor gene, and it’s highly likely to have a carcinogenic effect^24^. BRAF mutations are common in colorectal cancer and confers significant prognosis to advanced diseases^25^. Our study found that the difference in AQP9 expression was correlated with TP53 and BRAF mutants, which indicated a potential factor that might regulate AQP9 expression in breast and colon cancers. We also found that the promoter methylation level was lower in primary tumor tissues of COAD and LUSC than in their normal tissues. The reduced methylation level of AQP9 promoter in COAD may partly explain its increased expression. However, this couldn’t provide explanations for the down-regulated AQP9 expression in LUSC. This result suggested that AQP9 expression might also be affected by epigenetics to some extent, but the regulating effect varies from cancer to cancer.

Then, we further reported that discrepancies in AQP9 expression level had significant correlations with the prognosis of different tumors. Higher expression of AQP9 predicted worse prognosis for breast, colon and lung cancers. However, overexpression of AQP9 had a favorable prognosis in gastric cancer. AQP9 expression could impact the prognosis of patients with different lymph node status in breast, lung and gastric cancers indicating that AQP9 expression can be associated with tumor metastasis. Our findings were consistent with the previous study that high expression of AQP9 was associated with favorable OS in all gastric cancer patients including intestinal and diffused types ^26^, while high expression of AQP9 was accompanied by worse prognosis in patients with lymph node-negative breast cancer and was increased in higher SBR (Scarff–Bloom–Richardson) grades of all types of breast cancer in particular^27^. However, it may contradict and therefore discredit the results that AQP9 overexpression enhanced the cytotoxic response to 5-FU and promoted the activation of RAS through glycerol transport in CRC cells ^17^. It may suggest that the influence of AQP9 on the prognosis of cancer is not only related to its expression, but also related to its function of transport of various molecules and their influence on the specific tumor. Further researches are needed to determine whether AQP9 can be used to transport substances that are beneficial to the treatment of tumors so as to improve the clinical prognosis of patients with different cancers. AQP9 can be used to predict patients’ response to platinum-based chemotherapy and help to improve arsenic sensitivity in lung cancer^28^. It seemed that AQP9 could not only predict the clinical prognosis for tumor patients, but also be used as an indicator for the clinical treatment effect, providing directions for further improving the prognosis of patients. Taken together, our findings suggested that AQP9 had significant prognostic value in several distinct types of cancers and different AQP9 expression levels had different prognostic impact that might depend on the specific type of cancers. Further studies should be conducted to explore whether AQP9 can promote the uptake of specific drugs by tumor cells based on its own transport properties to enhance the sensitivity of chemotherapy.

In addition, the innovational aspect of this study clarified the significant correlations between AQP9 expression and various tumor-infiltrating immune cells in breast, colon, lung and gastric caners. Moreover, the correlations of AQP9 expression with the gene markers for immune cells indicated the potential function of AQP9 in regulating the tumor immunology of BRCA, COAD, LUAD, LUSC and STAD. Firstly, gene markers of M2 macrophage including CD163, VSIG4 and MS4A4A were moderately to very strongly correlated with AQP9 expression, while M1 macrophage markers such as PTGS2 and IRF5 were weakly associated with AQP9 expression. The M2 macrophages were closely related to worse prognosis in multiple types of malignant tumors^29^. And protumor macrophages have been reported to differentiate after interaction with tumor cells and participate in immunosuppression, invasion, and metastasis in different cancers^30,31^. These findings revealed the underlying regulatory role AQP9 might play in the polarization of TAMs, indicating AQP9 may be involved in the immunosuppression in the focused cancers. Then, for neutrophils, there was a significant correlation between AQP9 and CD11b in BRCA, COAD, LUAD, LUSC and STAD. As reported previously, neutrophils played a significant role in the inflammatory cell infiltrating levels in diverse types of cancer and could suppress CD8^+^T cell-mediated antitumor immune response^32^. CD11b was of significant importance in the detachment of neutrophil during chemotaxis and it could negatively regulate immune cell signaling pathways^33^. These findings suggested that AQP9 might affect tumor immune through neutrophils and the correlation with CD11b. In addition, AQP9 played a potentially important part in Tregs activation and T cell exhaustion induction. Overexpression of AQP9 had significantly positive correlations with the expression of markers for Treg cells and exhausted T cells, including FOXP3, CCR8, TGFB1, PD-1, CTLA4, TIM-3, LAG3 and GZMB in BRCA, COAD, LUAD, LUSC and STAD. FOXP3, an important marker in Treg cells, can inhibit cytotoxic T cells from attacking tumor cells^34^. TIM-3 was a vital protein on the surface of exhausted T cells^35^. These findings further revealed that AQP9 expression had a significantly strong relationship with tumor immune tolerance. Furthermore, our results also suggested that AQP9 expression was positively correlated with gene markers of DCs, such as HLA-DPB1, HLA-DRA and BDCA-4. It was reported that DCs could increase Treg cells and reduce cytotoxicity of CD8^+^T cells, thus promoting tumor metastasis^36^. Further studies are eagerly needed to illuminate whether AQP9 serves as a significant factor that mediates tumor metastasis through immune infiltrating cells. Moreover, AQP9 expression was observed to have significant correlations with most markers of several T-helper cells in BRCA, COAD, LUAD, LUSC and STAD. The results indicated a possible mechanism through which AQP9 regulated T cell functions. Although the effect of T cell-mediated immune responses has been well reported in tumors, the function of B cells in tumor progression has not been extensively studied^37^. Current studies have shown that tumorigenic mechanisms for infiltrating B cells include direct support for tumor growth and guidance for tumor-supporting immune cells^38^. Our findings suggested that AQP9 expression had a moderate negative correlation with infiltrating levels of B cells in STAD and the markers of B cells such as CD19 and CD79A had no significant correlations with AQP9 expression, which may partly explain the favorable prognosis correlated with increased AQP9 expression in gastric cancer. In conclusion, AQP9 expression had a significant impact on the regulation of immune infiltration levels and tumor-immune interaction in BRCA, COAD, LUAD, LUSC and STAD.

Furthermore, GO analysis demonstrated that genes co-expressed with AQP9 were significantly enriched in immune related gene sets in BRCA, COAD, LUAD, LUSC and STAD, which could be used as chemotherapeutic targets for cancer treatment^39^. KEGG analysis also revealed that genes co-expressed with AQP9 could be involved in immune related pathways in BRCA, COAD, LUAD, LUSC and STAD, which were also correlated to tumorigenesis or tumor progression^40^. The results indicated that AQP9 may regulate tumor immunity by affecting correlated genes and immune related pathways. We further investigated the mechanisms that AQP9 might participate in the regulation of tumor immunity through the network of the immune genes in correlation with AQP9. We found that immune related genes such as TLR8, Interleukin-7 (IL-7), Leptin (LEP), IL1RN had intimate relationships with AQP9 expression in breast, colon, lung and gastric cancers. Activation of TLR8 signaling could enhance anti-tumor immunity by reversing the inhibition of tumor-specific T cells in lung cancer^41^ and identify purified H. pylori RNA to induce inflammatory response in gastric cancer^42^. Moreover, TLR8 had positive correlation with IFN-β in breast cancer^43^ and could activate M1-like macrophages to disrupt platinum resistance in colorectal cancer^44^. IL-7 was a cytokine, primarily regarding its effects on T-cells and B-cells. It has been reported that IL-7 was up-regulated and could promote the proliferation of tumor-infiltrating lymphocytes in gastric and colorectal cancers^45^, while inhibit the proliferation of tumor cells in both lung and breast cancers^46,47^. Leptin could activate circulating monocytes, induce the secretion of TNFα and IL 6. It was reported to be associated with higher tumor stage in gastric cancer^48^ and it could also promote the invasion and migration of tumor cells in breast cancer by affecting TAMs^49^. Furthermore, ILIRN was prominent in regulating the release of interleukin-1beta. IL1RN polymorphism has been reported to promote the development of lung cancer through inflammatory response^50^. To sum up, AQP9 was correlated with these immune genes in tumor immune regulation. However, the underlying mechanism needed further study.

In summary, our study performed integrated analyses for the significance of AQP9 in prognosis and its potential role in tumor-immune interaction. Our results suggested that AQP9 expression had far-reaching effects in prognosis and immune cell infiltration in breast, colon, lung and gastric cancer patients. Compared with traditional research methods, bioinformatics has the advantages of large sample size, simplicity and low cost. However, there are also some limitations in our study. For example, our analyses are more based on the public databases, and the detailed molecular biological mechanisms in which AQP9 is involved to regulate the tumor infiltration need to be further validated. In the future research, more clinical information should be collected to further investigate the relationship between AQP9 expression and different pathologic characteristics of cancers. It should also be addressed through molecular biology techniques to confirm whether AQP9 can be used as a therapeutic target in the immunotherapy of cancers and the specific mechanism in which AQP9 affects the prognosis and immune infiltrates in breast, colon, lung and gastric cancers.

## Methods

### Gene expression analysis

Gene expression data including microarray experiments and the RNA-seq information were obtained from the GEO (Gene Expression Omnibus) and TCGA (The Cancer Genome Atlas) databases and analyzed in Oncomine and TIMER databases. Oncomine (http://www.oncomine.org) is a powerful online cancer database that has identified the genes and pathways through a large collection of cancer gene expression microarrays^51^. The mRNA expression level of AQP9 in diverse cancer types was examined via Oncomine database and the results were determined according to the following thresholds: P-value < 0.001, fold change > 1.5, and gene ranking of all. The mRNA expression level of AQP9 in different cancers was further evaluated by using the RNA-seq data across all TCGA tumors with statistical significance evaluated using Wilcoxon test in TIMER (P-value < 0.05). The Human Protein Atlas (HPA) provided researchers with a convenient and valuable platform for investigating protein expression and localization in human cells and tissues^52^. The protein expression level of AQP9 was detected by using HPA database (https://www.proteinatlas.org/). The LinkedOmics database (http://www.linkedomics.org) is a multi-omics database that includes clinical data for a total of 32 cancer types from TCGA^53^. The correlation between the AQP9 expression level and the gene mutation level in different cancers was analyzed via LinkedOmics database with a significance of P-value < 0.05. UALCAN (http://ualcan.path.uab.edu/) is an online database for facilitating the analyses of gene expression and survival in multiple malignances^54^. The promoter methylation expression level of AQP9 between normal and cancer tissue was analyzed by using UALCAN according to the online instruction (P-value < 0.05).

### The prognostic value of AQP9 analysis

Kaplan–Meier plotter (http://kmplot.com/analysis/) can evaluate the influence of 54,675 genes on survival rate across 10,461 samples from diverse cancer types^55^. Prognostic information of patients with breast (n = 6234), ovarian (n = 2190), Lung (n = 3452), and gastric (n = 1440) cancers were sourced from GEO, TCGA and EGA (The European Genome-phenome Archive) databases and studied in the Kaplan–Meier Plotter database. The correlation between the AQP9 expression and the specific clinical characters were also analyzed in Kaplan–Meier plotter database. Log rank P-value and hazard ratio (HR) with 95% confidence intervals were generated and displayed on the webpage (log-rank P < 0.05). PrognoScan (http://gibk21.bse.kyutech.ac.jp/PrognoScan/index.html) provides a powerful platform, which can be used to determine the significant prognostic value of the gene expression, such as overall survival (OS), disease-specific survival (DSS), disease-free survival (DFS) and distant metastasis-free survival (DMFS) across publicly available cancer microarrays^56^. The correlations of AQP9 expression with the survival rate in diverse cancers were examined through the PrognoScan with a significant threshold of corrected P-value < 0.05.

### Correlation analysis between AQP9 expression and immune infiltrating levels

TIMER (https://cistrome.shinyapps.io/timer/) is a comprehensive resource platform which can access the systematic analysis of immune infiltrates in various types of cancers^57^. AQP9 gene was analyzed in the gene module to explore the correlation of AQP9 expression with the immune infiltrating abundance. Furthermore, the correlation module was also applied to determine the correlations of AQP9 expression with gene marker sets of different immune infiltrating cells, including T cells, B cells, monocytes, TAMs, macrophages, neutrophils, natural killer (NK) cells, DCs, different T-helper cells, Tregs, and exhausted T cells. These representative gene markers were referenced in previous reports^58^ and the CellMarker database (http://biocc.hrbmu.edu.cn/CellMarker/)^59^. GEPIA (http://gepia.cancer-pku.cn/) was also utilized to investigate the gene expression correlation for given sets by using TCGA data (P-value < 0.05).

### GO analysis, KEGG analysis and the network construction of genes co-expressed with AQP9

The differentially expressed genes in significant correlation with AQP9 were obtained from the LinkedOmics database with an adjusted p-value (FDR < 0.05). The gene ontology (GO) enrichment analysis and the KEGG analysisyy^60^ of the genes co-expressed with AQP9 were generated from LinkedOmics database. The network of the immune-related genes in correlation with AQP9 was constructed in STRING (http://string-db.org/) and Cytoscape.

### Statistical analyses

The expression data generated in Oncomine and TIMER database are displayed with P-values. The survival data of PrognoScan, Kaplan–Meier plotter, and the results of GEPIA database are evaluated by HR and Cox P or P-values from a log-rank test. Spearman’s correlation was utilized to assess the correlation of gene expression, and the correlation intensity was determined under the guidance of absolute value: 0.00–0.19 for “very weak”, 0.20–0.39 for “weak”, 0.40–0.59 for “moderate”, 0.60–0.79 for “strong” and 0.80–1.0 for “very strong.” The gene expression levels were calculated and displayed with log2 RSEM in TIMER database. All statistical tests were two-tailed and P < 0.05 was considered statistically significant.

## Supplementary information

Supplementary Information

## Data Availability

All data generated or analyzed during this study are included in this article (and its Supplementary Information files).
