# Supplementary material for: Integrated analysis identifies AQP9 correlates with immune infiltration and acts as a prognosticator in multiple cancers
Source: Sci Rep. 2020 Nov 27;10:20795. doi: 10.1038/s41598-020-77657-z (PMC7699650; doi:10.1038/s41598-020-77657-z)
Supplement: Supplementary file 1 — Supplementary Information [file 41598_2020_77657_MOESM1_ESM.pdf]

Supplementary information

Integrated analysis identifies AQP9 correlates with immune infiltration and acts as a prognosticator in multiple cancers

Xiaohong Liu[1], Qian Xu[1], Zijing Li[1] & Bin Xiong[1,2,3]*

Author affiliations:
1Department of Gastrointestinal Surgery & Department of Gastric and Colorectal Surgical Oncology, Zhongnan Hospital of Wuhan University, Wuhan 430071, People's Republic of China.
2Hubei Key Laboratory of Tumor Biological Behaviors, Wuhan 430071, People's Republic of China.
3Hubei Cancer Clinical Study Center, Wuhan 430071, People's Republic of China.
***Correspondence:**
Bin Xiong (binxiong1961@whu.edu.cn)

Supplementary information:5 figures and 6 tables

Normal

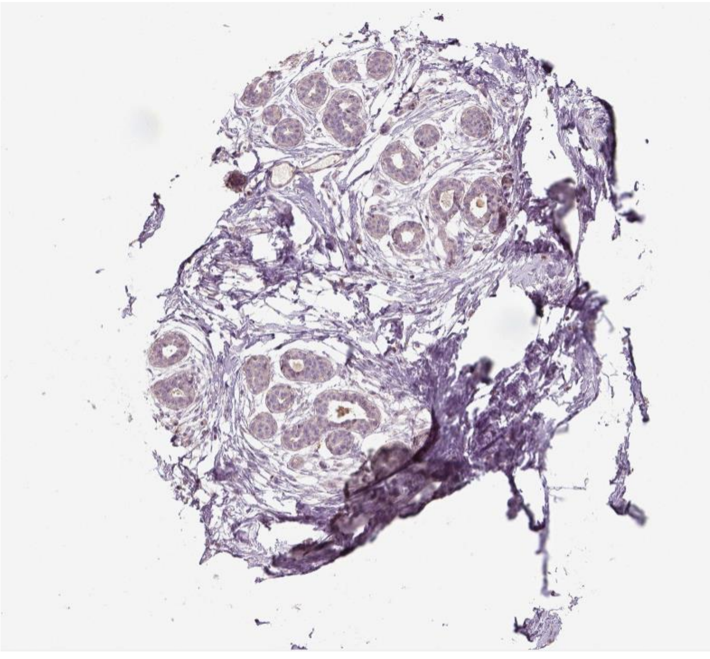

Breast (T-04000)
Normal tissue, NOS (M-00100)
Antibody:CAB075684
Female, age 45
Patient id: 3544
Glandular cells
Staining: Not detected
Intensity: Weak
Quantity: <25%
Location: Cytoplasmic/membranous

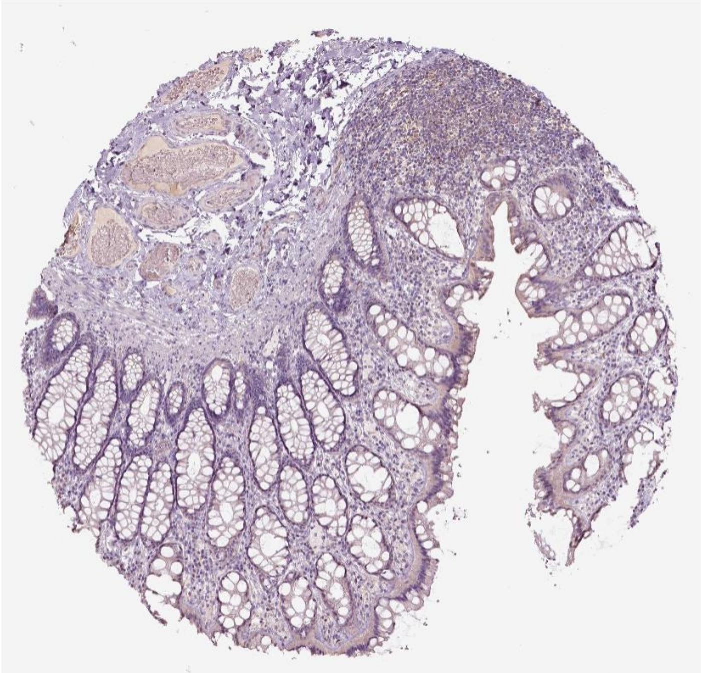

Colon (T-67000)
Normal tissue, NOS (M-00100)
Antibody: CAB075684
Male, age 47
Patient id: 1857
Glandular cells
Staining: Not detected
Intensity: Weak
Quantity: <25%
Location: Cytoplasmic/membranous

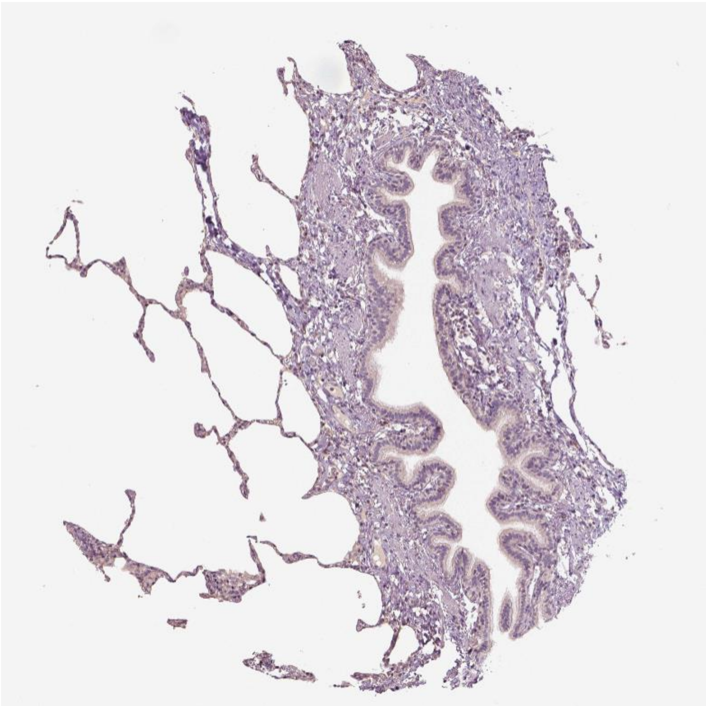

Lung (T-28000)
Normal tissue, NOS (M-00100)
Antiboody:CAB075684
Female, age 57
Patient id: 1678
Macrophages
Staining: Not detected
Intensity: Weak
Quantity: <25%
Location: Cytoplasmic/membranous

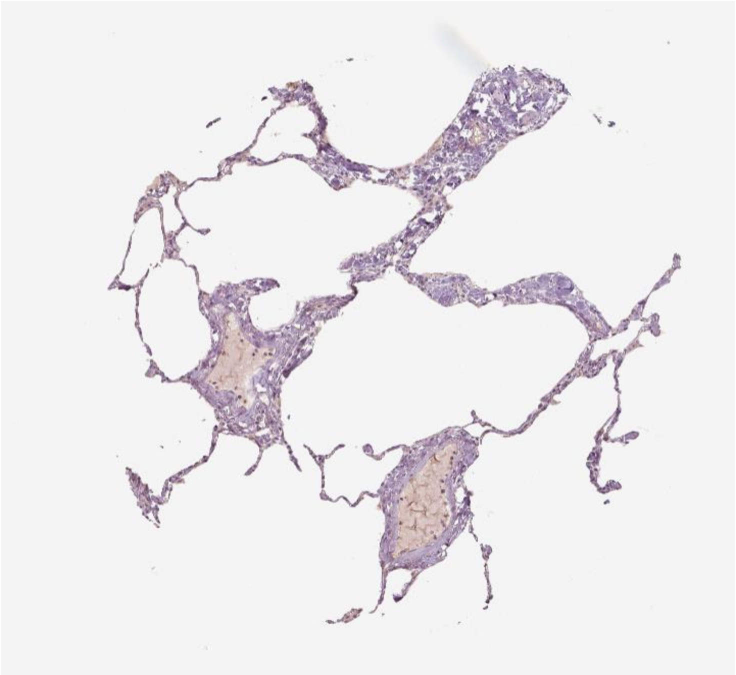

Lung (T-28000)
Normal tissue, NOS (M-00100)
Antiboody: CAB075684
Male, age 65
Patient id: 1470
Pneumocytes
Staining: Not detected
Intensity: Negative
Quantity: None
Location: None

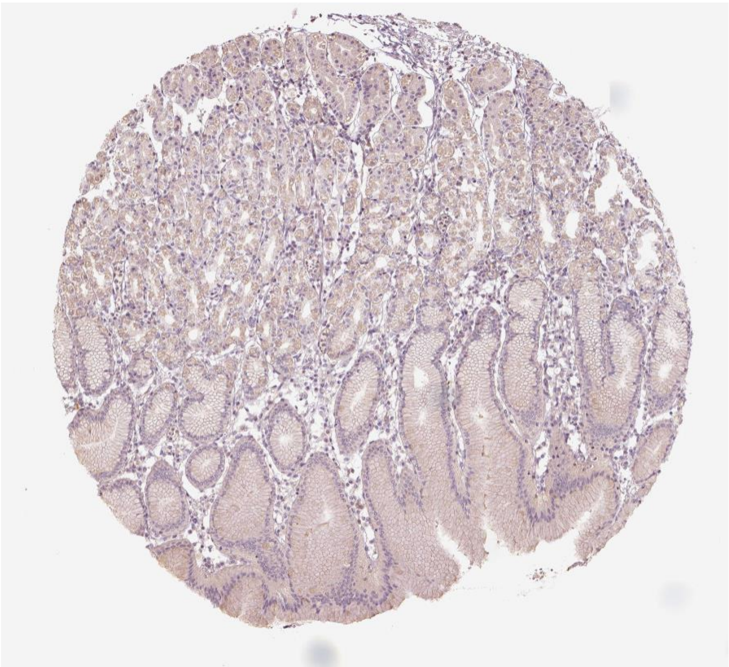

Stomach (T-63000)
Normal tissue, NOS (M-00100)
Antibody: CAB075684
Female, age 57
Patient id: 1467
Glandular cells
Staining: Not detected
Intensity: Weak
Quantity: <25%
Location: Cytoplasmic/membranous

Cancer

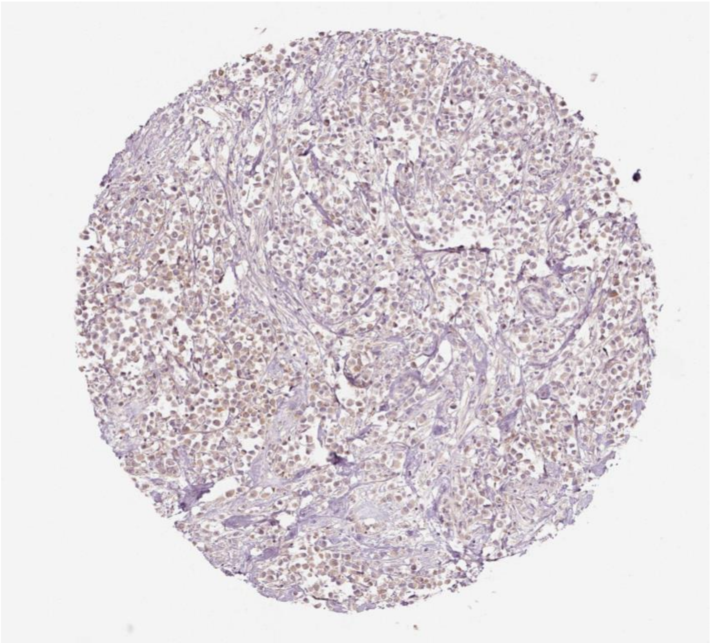

Breast (T-04000)
Duct carcinoma (M-85003)
Antibody: CAB075684
Female, age 61
Patient id: 1910
Tumor cells
Staining: Low
Intensity: Moderate
Quantity: <25%
Location: Cytoplasmic/membranous

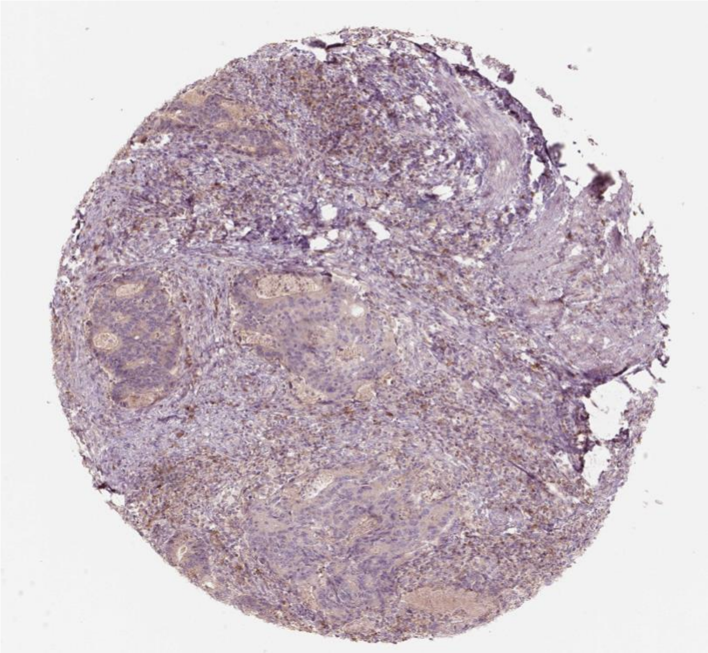

Colon (T-67000)
Adenocarcinoma, NOS (M-81403)
Antibody: CAB075684
Male, age 67
Patient id: 4720
Tumor cells
Staining: Not detected
Intensity: Weak
Quantity: <25%
Location: Cytoplasmic/membranous

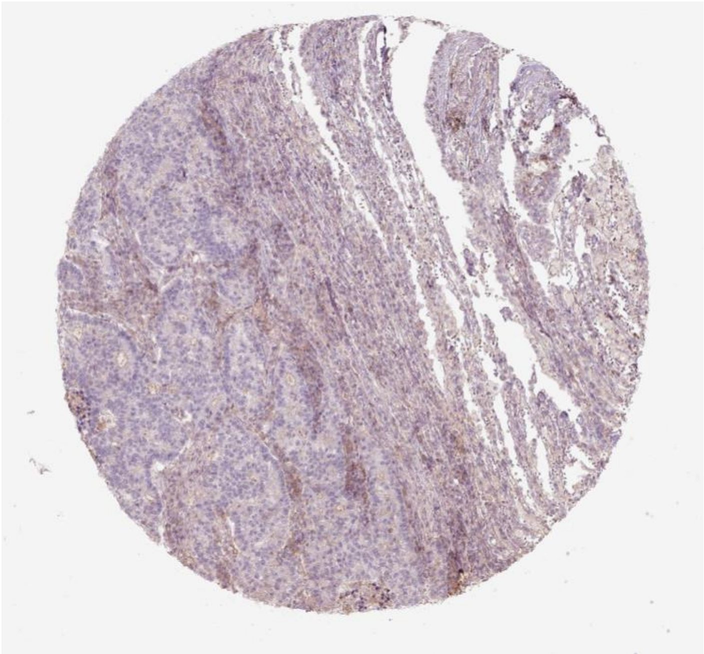

Lung (T-28000)
Adenocarcinoma, NOS (M-81403)
Antiboody:CAB075684
Male, age 57
Patient id: 4923
Tumor cells
Staining: Not detected
Intensity: Weak
Quantity: <25%
Location: Cytoplasmic/membranous

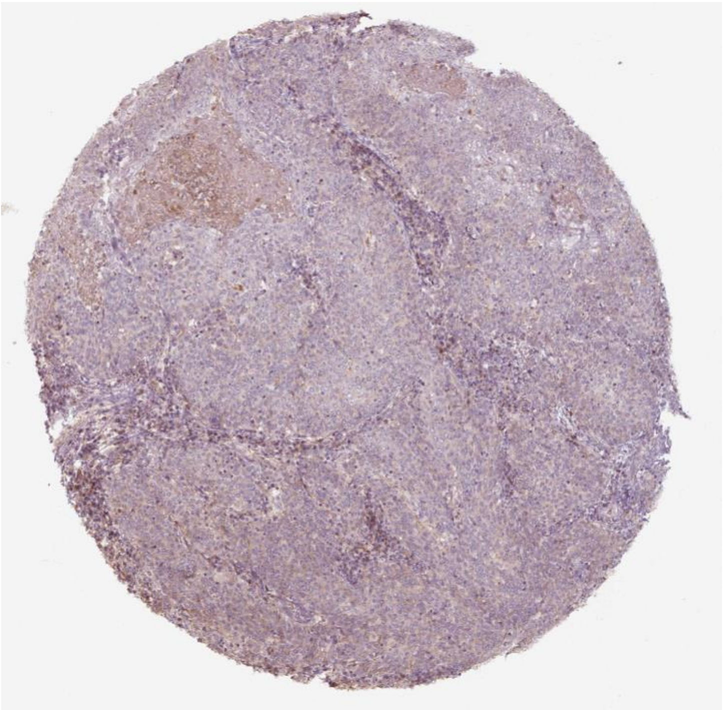

Lung (T-28000)
Squamous cell carcinoma, NOS (M-80703)
Antibody:CAB075684
Male, age 82
Patient id: 4488
Tumor cells
Staining: Low
Intensity: Weak
Quantity: 75%-25%
Location: Cytoplasmic/membranous

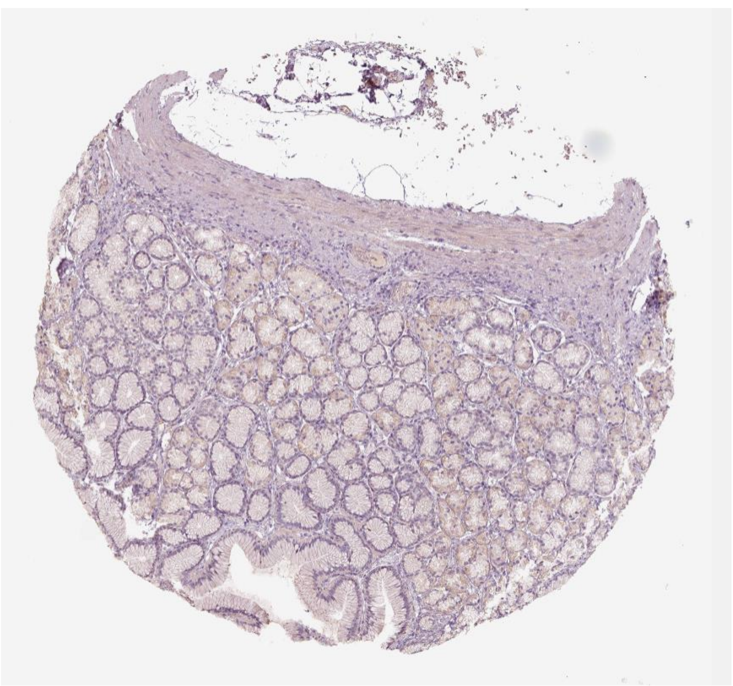

Stomach (T-63000)
Adenocarcinoma, NOS (M-81403)
Antibody: CAB075684
Female, age 65
Patient id: 2326
Tumor cells
Staining: Not detected
Intensity: Weak
Quantity: <25%
Location: Cytoplasmic/membranous

Supplementary Figure 1. AQP9 expression of protein level in the human protein atlas database.

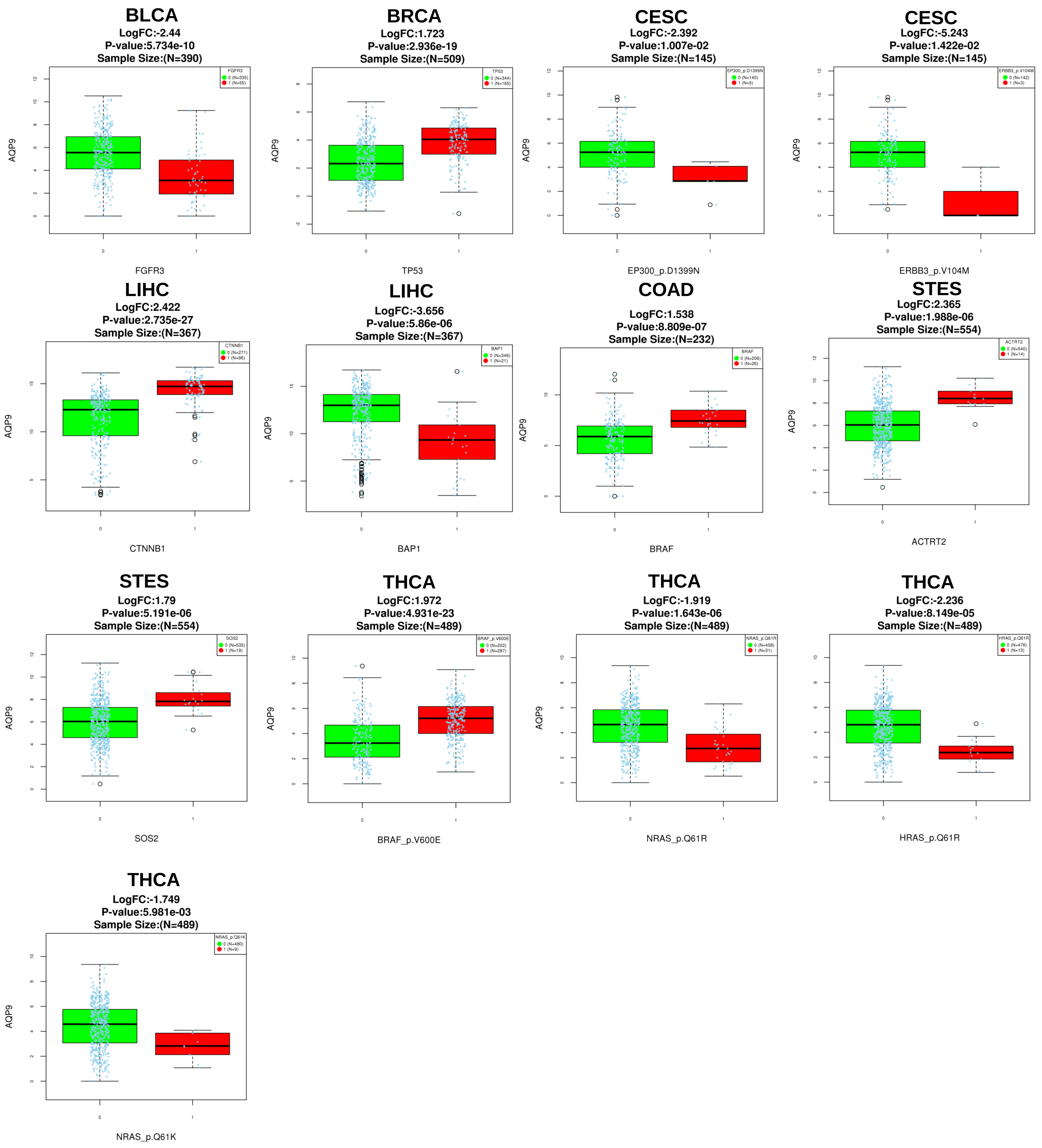

Supplementary Figure 2. Relevance of AQP9 expression and gene mutation in different cancers in Linkedomics database. 0= Wild type, 1= Mutation.

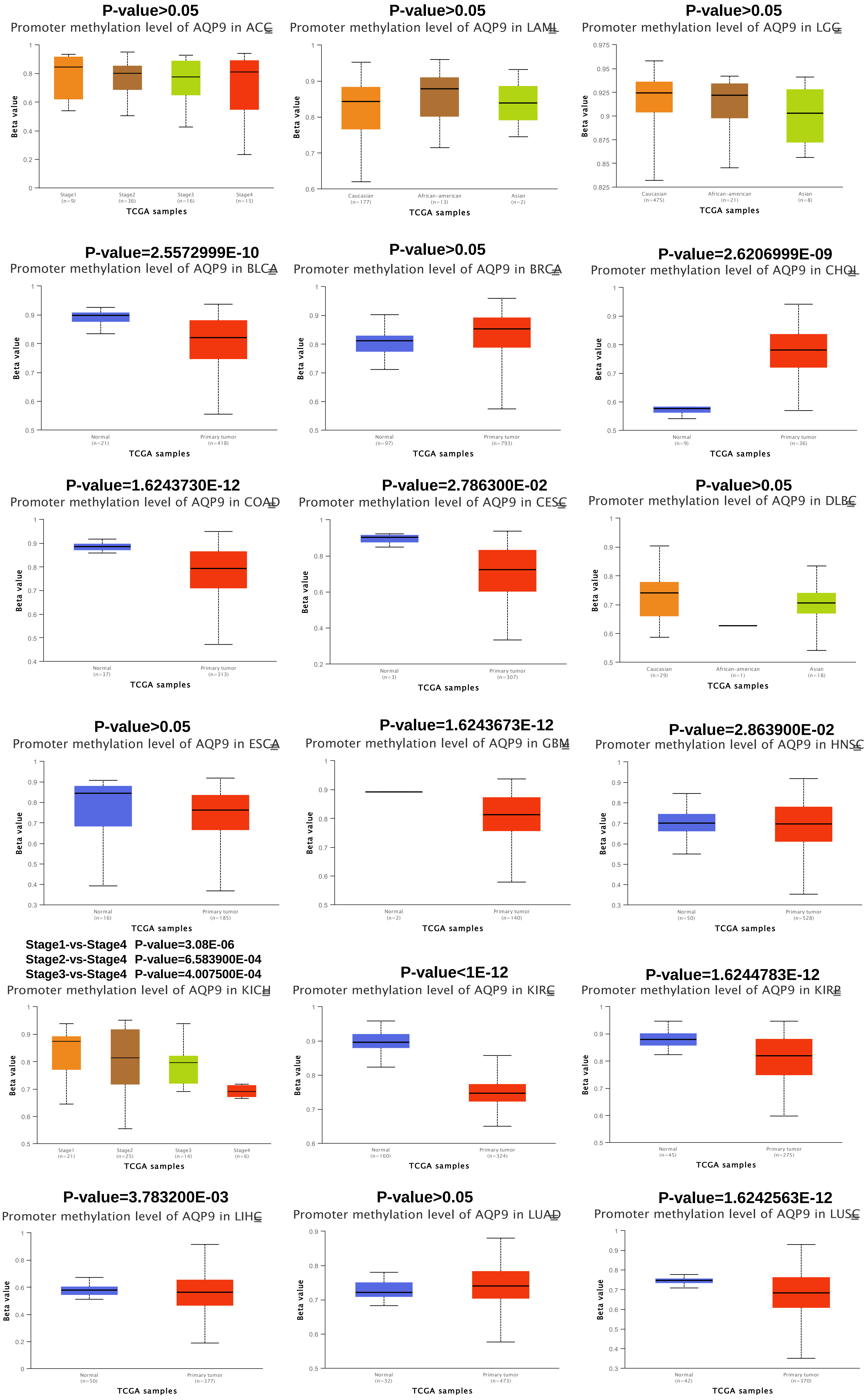

P-value>0.05
Promoter methylation level of AQP9 in ACC
Beta value
Stage1 (n=9)
Stage2 (n=36)
Stage3 (n=16)
Stage4 (n=15)
TCGA samples
P-value>0.05
Promoter methylation level of AQP9 in LAML
Beta value
Caucasian (n=177)
African-american (n=13)
Asian (n=2)
TCGA samples
P-value>0.05
Promoter methylation level of AQP9 in LGG
Beta value
Caucasian (n=475)
African-american (n=21)
Asian (n=8)
TCGA samples
P-value=2.5572999E-10
Promoter methylation level of AQP9 in BLCA
Beta value
Normal (n=21)
Primary tumor (n=418)
TCGA samples
P-value>0.05
Promoter methylation level of AQP9 in BRCA
Beta value
Normal (n=97)
Primary tumor (n=793)
TCGA samples
P-value=2.6206999E-09
Promoter methylation level of AQP9 in CHOL
Beta value
Normal (n=9)
Primary tumor (n=36)
TCGA samples
P-value=1.6243730E-12
Promoter methylation level of AQP9 in COAD
Beta value
Normal (n=37)
Primary tumor (n=313)
TCGA samples
P-value=2.786300E-02
Promoter methylation level of AQP9 in CESC
Beta value
Normal (n=3)
Primary tumor (n=307)
TCGA samples
P-value>0.05
Promoter methylation level of AQP9 in DLBC
Beta value
Caucasian (n=29)
African-american (n=1)
Asian (n=18)
TCGA samples
P-value>0.05
Promoter methylation level of AQP9 in ESCA
Beta value
Normal (n=16)
Primary tumor (n=185)
TCGA samples
P-value=1.6243673E-12
Promoter methylation level of AQP9 in GBM
Beta value
Normal (n=2)
Primary tumor (n=140)
TCGA samples
P-value=2.863900E-02
Promoter methylation level of AQP9 in HNSC
Beta value
Normal (n=50)
Primary tumor (n=528)
TCGA samples
Stage1-vs-Stage4 P-value=3.08E-06
Stage2-vs-Stage4 P-value=6.583900E-04
Stage3-vs-Stage4 P-value=4.007500E-04
Promoter methylation level of AQP9 in KICH
Beta value
Stage1 (n=21)
Stage2 (n=25)
Stage3 (n=14)
Stage4 (n=6)
TCGA samples
P-value<1E-12
Promoter methylation level of AQP9 in KIRC
Beta value
Normal (n=160)
Primary tumor (n=324)
TCGA samples
P-value=1.6244783E-12
Promoter methylation level of AQP9 in KIRP
Beta value
Normal (n=45)
Primary tumor (n=275)
TCGA samples
P-value=3.783200E-03
Promoter methylation level of AQP9 in LIHC
Beta value
Normal (n=50)
Primary tumor (n=377)
TCGA samples
P-value>0.05
Promoter methylation level of AQP9 in LUAD
Beta value
Normal (n=32)
Primary tumor (n=473)
TCGA samples
P-value=1.6242563E-12
Promoter methylation level of AQP9 in LUSC
Beta value
Normal (n=42)
Primary tumor (n=370)
TCGA samples

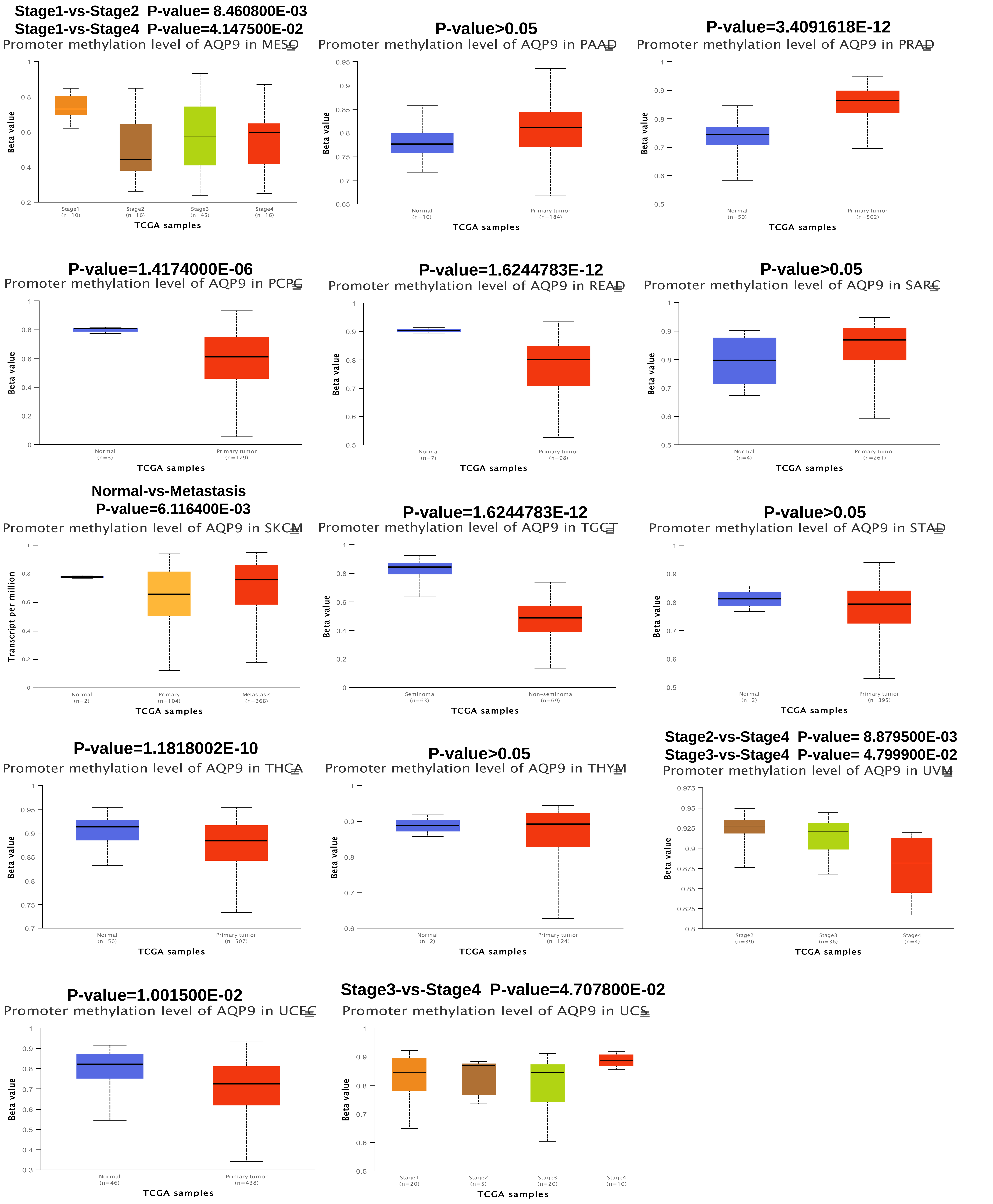

Supplementary Figure 3. AQP9 promoter methylation levels in different cancers in UALCAN database.

CANCER TYPE	DATASET	ENDPOINT	N	HR [95% CI]	Cox P
Bladder cancer	GSE5287	Overall Survival	30	1.05 [0.67 - 1.65]	0.829188
	GSE13507	Overall Survival	165	1.52 [1.15 - 2.00]	0.00285584
	GSE13507	Disease Specific Survival	165	1.57 [1.10 - 2.23]	0.0123259
Blood cancer	GSE12417-GPL96	Overall Survival	163	0.86 [0.69 - 1.08]	0.207114
	GSE12417-GPL570	Overall Survival	79	1.01 [0.84 - 1.21]	0.924842
	GSE5122	Overall Survival	58	1.27 [0.96 - 1.70]	0.097749
	GSE8970	Overall Survival	34	0.92 [0.72 - 1.18]	0.519701
	GSE4475	Overall Survival	158	0.81 [0.44 - 1.51]	0.517671
	E-TABM-346	Overall Survival	53	1.29 [0.95 - 1.75]	0.0977444
	E-TABM-346	Event Free Survival	53	1.16 [0.87 - 1.55]	0.303276
	GSE16131-GPL96	Overall Survival	180	1.16 [0.96 - 1.39]	0.12696
	GSE2658	Disease Specific Survival	559	1.12 [0.80 - 1.56]	0.520446
Brain cancer	GSE4271-GPL96	Overall Survival	77	1.02 [0.86 - 1.21]	0.806148
	GSE7696	Overall Survival	70	0.89 [0.57 - 1.38]	0.595075
	MGH-glioma	Overall Survival	50	0.65 [0.19 - 2.23]	0.48962
	GSE4412-GPL96	Overall Survival	74	1.35 [1.09 - 1.66]	0.00598368
	GSE16581	Overall Survival	67	1.73 [0.66 - 4.51]	0.262573
Breast cancer	GSE19615	Distant Metastasis Free Survival	115	0.93 [0.50 - 1.72]	0.816955
	GSE3143	Overall Survival	158	1.28 [0.85 - 1.92]	0.23413
	GSE7849	Disease Free Survival	76	0.67 [0.34 - 1.32]	0.249021
	GSE12276	Relapse Free Survival	204	1.18 [1.05 - 1.31]	0.00351786
	GSE6532-GPL570	Relapse Free Survival	87	1.03 [0.64 - 1.64]	0.904943
	GSE6532-GPL570	Distant Metastasis Free Survival	87	1.03 [0.64 - 1.64]	0.904943
	GSE9195	Relapse Free Survival	77	2.25 [0.51 - 3.94]	0.283826
	GSE9195	Distant Metastasis Free Survival	77	0.98 [0.18 - 5.46]	0.984006
	GSE12093	Distant Metastasis Free Survival	136	0.76 [0.42 - 1.36]	0.351489
	GSE11121	Distant Metastasis Free Survival	200	1.33 [1.05 - 1.69]	0.0183157
	GSE1378	Relapse Free Survival	60	1.74 [1.22 - 2.49]	0.00237674
	GSE1379	Relapse Free Survival	60	1.59 [1.19 - 2.12]	0.00169882
	GSE9893	Overall Survival	155	0.86 [0.60 - 1.23]	0.402113
	GSE2034	Distant Metastasis Free Survival	286	1.03 [0.86 - 1.22]	0.767527
	GSE1456-GPL96	Overall Survival	159	1.51 [1.13 - 2.02]	0.00493075
	GSE1456-GPL96	Relapse Free Survival	159	1.77 [1.35 - 2.31]	3.15E-05
	GSE1456-GPL96	Disease Specific Survival	159	1.89 [1.39 - 2.59]	6.20E-05
	GSE7378	Disease Free Survival	54	0.61 [0.19 - 1.95]	0.409919
	E-TABM-158	Overall Survival	117	0.72 [0.43 - 1.20]	0.211833
	E-TABM-158	Distant Metastasis Free Survival	117	0.65 [0.34 - 1.26]	0.204427
	E-TABM-158	Relapse Free Survival	117	0.72 [0.43 - 1.20]	0.211833
	E-TABM-158	Disease Specific Survival	117	0.53 [0.26 - 1.06]	0.0735835
	GSE3494-GPL96	Disease Specific Survival	236	1.39 [1.09 - 1.77]	0.00893436
	GSE4922-GPL96	Disease Free Survival	249	1.33 [1.10 - 1.62]	0.00331805
	GSE2990	Relapse Free Survival	62	0.95 [0.69 - 1.32]	0.770513
	GSE2990	Distant Metastasis Free Survival	125	0.76 [0.29 - 2.01]	0.582082
	GSE2990	Relapse Free Survival	125	0.83 [0.40 - 1.72]	0.616731
	GSE2990	Distant Metastasis Free Survival	54	0.96 [0.65 - 1.42]	0.832637
	GSE7390	Overall Survival	198	1.26 [1.06 - 1.50]	0.0101399
	GSE7390	Relapse Free Survival	198	1.20 [1.05 - 1.36]	0.00698317
	GSE7390	Distant Metastasis Free Survival	198	1.22 [1.04 - 1.44]	0.0156143
Colorectal cancer	GSE12945	Disease Free Survival	51	4.95 [0.13 - 5.92]	0.388637
	GSE12945	Overall Survival	62	4.97 [0.71 - 3.62]	0.105056
	GSE17536	Disease Free Survival	145	1.66 [1.05 - 2.61]	0.0305355
	GSE17536	Disease Specific Survival	177	1.18 [0.80 - 1.73]	0.401005
	GSE17536	Overall Survival	177	1.10 [0.79 - 1.53]	0.583334
	GSE14333	Disease Free Survival	226	1.12 [0.96 - 1.30]	0.160881
	GSE17537	Disease Specific Survival	49	0.64 [0.24 - 1.71]	0.371421
	GSE17537	Overall Survival	55	1.11 [0.65 - 1.91]	0.704764
	GSE17537	Disease Free Survival	55	0.92 [0.49 - 1.72]	0.795933
Esophagus cancer	GSE11595	Overall Survival	34	0.48 [0.25 - 0.95]	0.0363522
Eye cancer	GSE22138	Distant Metastasis Free Survival	63	0.00 [0.00 - 1.50]	0.116157
Head and neck cancer	GSE2837	Relapse Free Survival	28	1.73 [0.50 - 5.95]	0.384881
Lung cancer	jacob-00182-CANDF	Overall Survival	82	0.79 [0.46 - 1.36]	0.394001
	jacob-00182-HLM	Overall Survival	79	1.00 [0.64 - 1.57]	0.993461
	jacob-00182-MSK	Overall Survival	104	2.60 [1.40 - 4.82]	0.0024534
	GSE13213	Overall Survival	117	0.95 [0.77 - 1.18]	0.666353
	GSE31210	Overall Survival	204	1.55 [0.98 - 2.43]	0.0593059
	GSE31210	Relapse Free Survival	204	1.42 [1.01 - 2.00]	0.0462311
	jacob-00182-UM	Overall Survival	178	1.15 [0.88 - 1.50]	0.304842
	GSE11117	Overall Survival	41	0.79 [0.60 - 1.05]	0.104072
	GSE3141	Overall Survival	111	1.16 [0.93 - 1.45]	0.193924
	GSE14814	Overall Survival	90	0.86 [0.41 - 1.84]	0.704447
	GSE14814	Disease Specific Survival	90	0.88 [0.37 - 2.09]	0.773489
	GSE8894	Relapse Free Survival	138	1.26 [1.02 - 1.56]	0.0306346
	GSE4573	Overall Survival	129	0.95 [0.76 - 1.18]	0.645071
	GSE17710	Overall Survival	56	0.86 [0.70 - 1.06]	0.159973
	GSE17710	Relapse Free Survival	56	0.88 [0.72 - 1.07]	0.20114
Ovarian cancer	GSE9891	Overall Survival	278	0.95 [0.82 - 1.10]	0.491562
	DUKE-OC	Overall Survival	133	1.09 [0.90 - 1.33]	0.372691
	GSE8841	Overall Survival	81	0.54 [0.29 - 1.00]	0.0512355
	GSE26712	Disease Free Survival	185	1.15 [0.91 - 1.45]	0.231907
	GSE26712	Overall Survival	185	1.15 [0.89 - 1.48]	0.293319
	GSE17260	Progression Free Survival	110	1.08 [0.95 - 1.23]	0.256993
	GSE17260	Overall Survival	110	1.10 [0.93 - 1.30]	0.253602
	GSE14764	Overall Survival	80	0.90 [0.68 - 1.20]	0.47078
Prostate cancer	GSE16560	Overall Survival	281	1.00 [0.84 - 1.20]	0.976018
Skin cancer	GSE19234	Overall Survival	38	1.07 [0.73 - 1.58]	0.724981
Soft tissue cancer	GSE30929	Distant Recurrence Free Survival	140	0.48 [0.23 - 1.02]	0.0575244

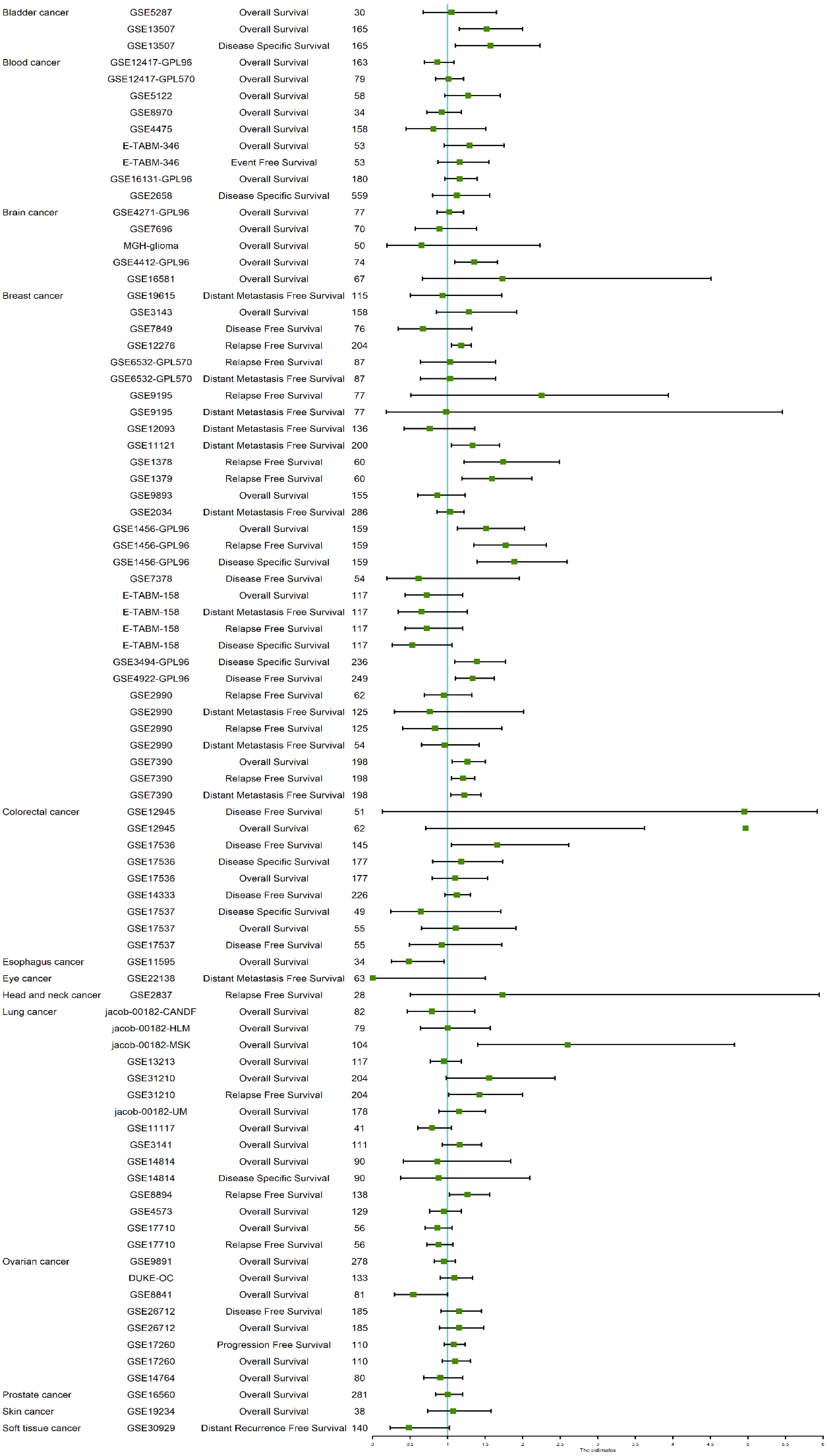

Supplementary Figure 4. Relation between AQP9 expression and patient progonsis of different cancers in Prognoscan database.

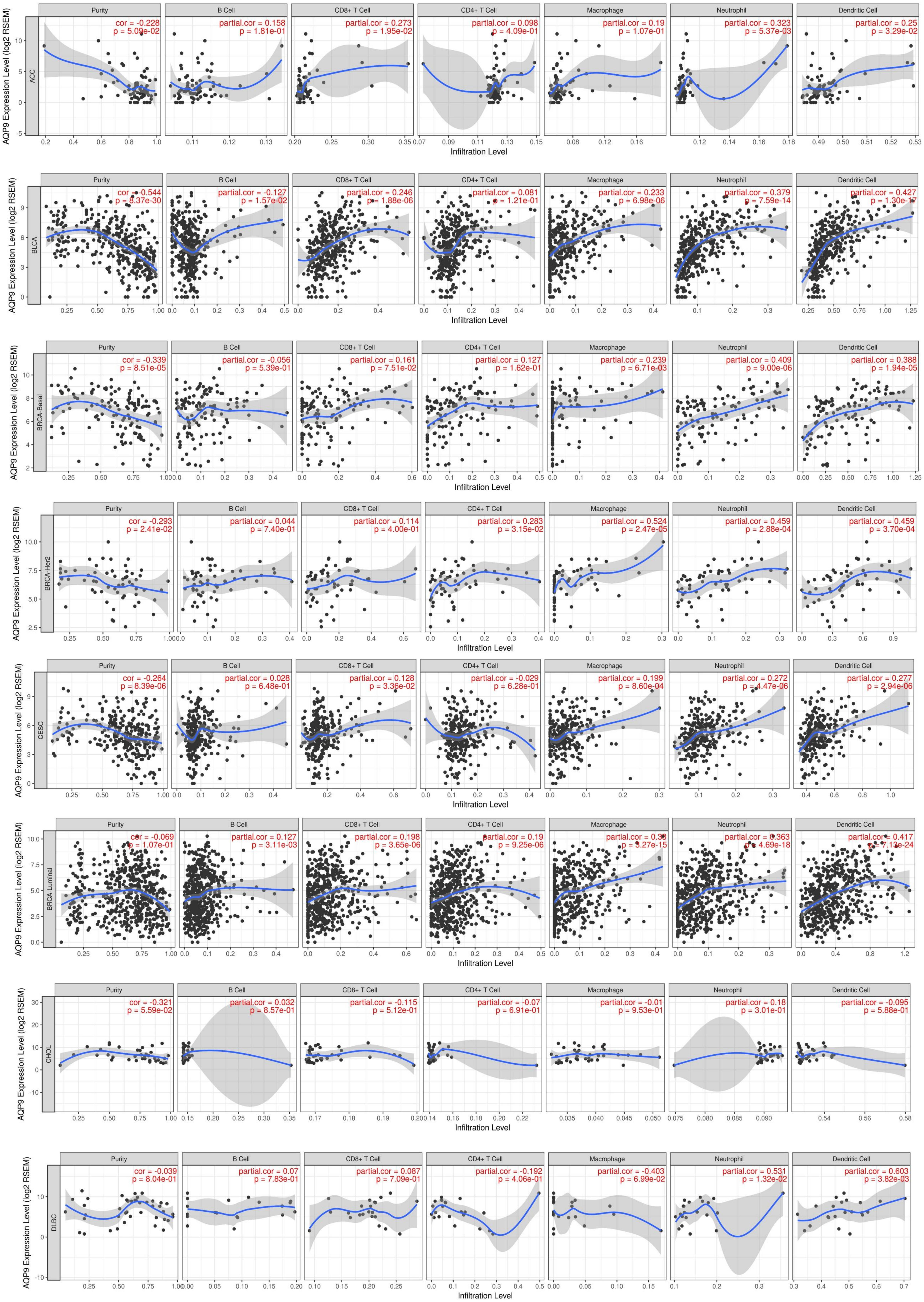
AQP9 Expression Level (log2 RSEM)
Infiltration Level
Purity
B Cell
CD8+ T Cell
CD4+ T Cell
Macrophage
Neutrophil
Dendritic Cell
ACC
cor = -0.228 p = 5.09e-02
partial.cor = 0.158 p = 1.81e-01
partial.cor = 0.273 p = 1.95e-02
partial.cor = 0.098 p = 4.09e-01
partial.cor = 0.19 p = 1.07e-01
partial.cor = 0.323 p = 5.37e-03
partial.cor = 0.25 p = 3.29e-02
BLCA
cor = -0.544 p = 8.37e-30
partial.cor = -0.127 p = 1.57e-02
partial.cor = 0.246 p = 1.88e-06
partial.cor = 0.081 p = 1.21e-01
partial.cor = 0.233 p = 6.98e-06
partial.cor = 0.379 p = 7.59e-14
partial.cor = 0.427 p = 1.30e-17
BRCA-Basal
cor = -0.339 p = 8.51e-05
partial.cor = -0.056 p = 5.39e-01
partial.cor = 0.161 p = 7.51e-02
partial.cor = 0.127 p = 1.62e-01
partial.cor = 0.239 p = 6.71e-03
partial.cor = 0.409 p = 9.00e-06
partial.cor = 0.388 p = 1.94e-05
BRCA-Her2
cor = -0.293 p = 2.41e-02
partial.cor = 0.044 p = 7.40e-01
partial.cor = 0.114 p = 4.00e-01
partial.cor = 0.283 p = 3.15e-02
partial.cor = 0.524 p = 2.47e-05
partial.cor = 0.459 p = 2.88e-04
partial.cor = 0.459 p = 3.70e-04
CESC
cor = -0.264 p = 8.39e-06
partial.cor = 0.028 p = 6.48e-01
partial.cor = 0.128 p = 3.36e-02
partial.cor = -0.029 p = 6.28e-01
partial.cor = 0.199 p = 8.60e-04
partial.cor = 0.272 p = 4.47e-06
partial.cor = 0.277 p = 2.94e-06
BRCA-Luminal
cor = -0.069 p = 1.07e-01
partial.cor = 0.127 p = 3.11e-03
partial.cor = 0.198 p = 3.65e-06
partial.cor = 0.19 p = 9.25e-06
p = 5.27e-15
partial.cor = 0.363 p = 4.69e-18
partial.cor = 0.417 p = 7.13e-24
CHOL
cor = -0.321 p = 5.59e-02
partial.cor = 0.032 p = 8.57e-01
partial.cor = -0.115 p = 5.12e-01
partial.cor = -0.07 p = 6.91e-01
partial.cor = -0.01 p = 9.53e-01
partial.cor = 0.18 p = 3.01e-01
partial.cor = -0.095 p = 5.88e-01
DLBC
cor = -0.039 p = 8.04e-01
partial.cor = 0.07 p = 7.83e-01
partial.cor = 0.087 p = 7.09e-01
partial.cor = -0.192 p = 4.06e-01
partial.cor = -0.403 p = 6.99e-02
partial.cor = 0.531 p = 1.32e-02
partial.cor = 0.603 p = 3.82e-03

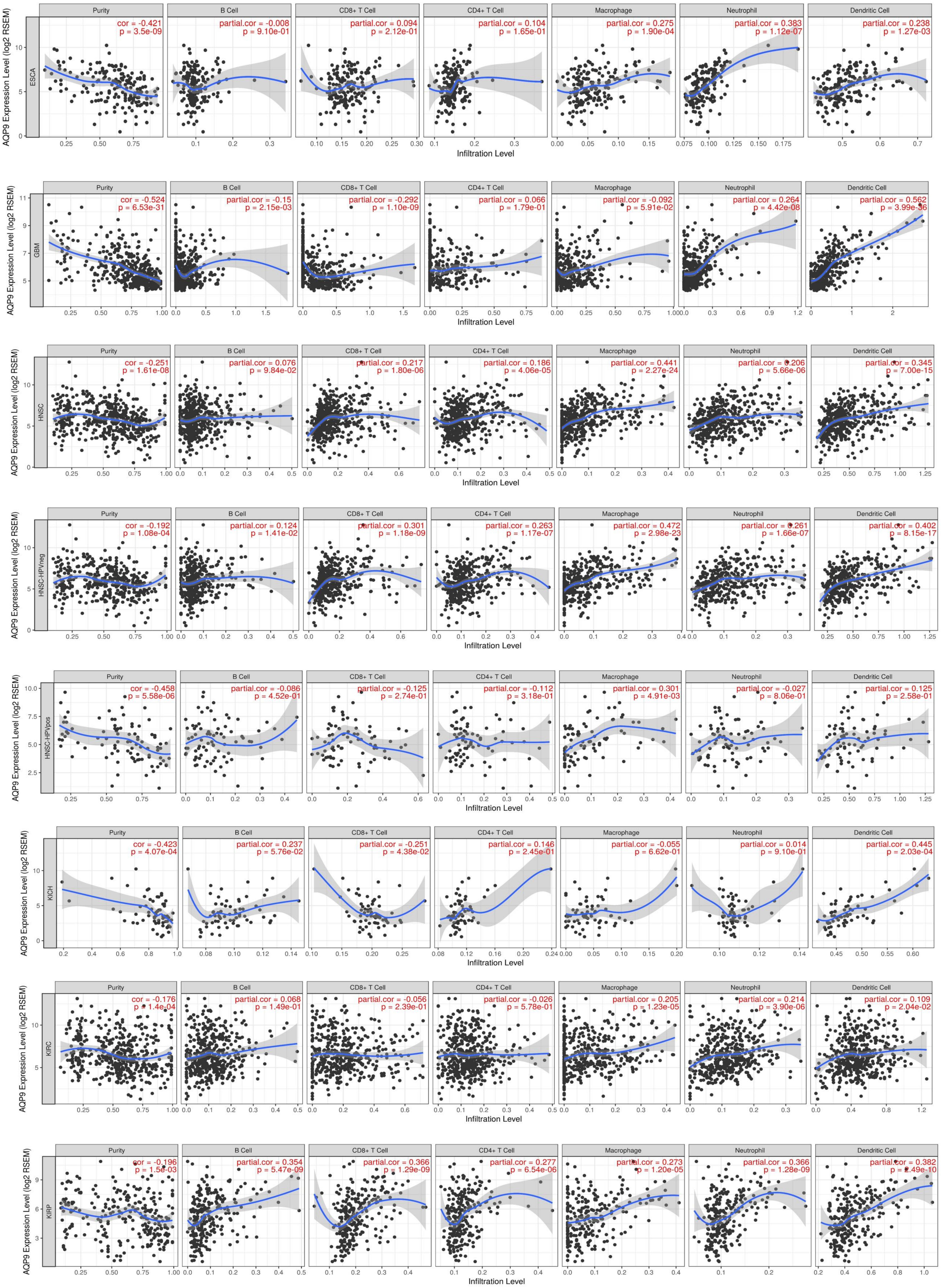
AQP9 Expression Level (log2 RSEM)
Infiltration Level
Purity
B Cell
CD8+ T Cell
CD4+ T Cell
Macrophage
Neutrophil
Dendritic Cell
ESCA
cor = -0.421 p = 3.5e-09
partial.cor = -0.008 p = 9.10e-01
partial.cor = 0.094 p = 2.12e-01
partial.cor = 0.104 p = 1.65e-01
partial.cor = 0.275 p = 1.90e-04
partial.cor = 0.383 p = 1.12e-07
partial.cor = 0.238 p = 1.27e-03
GBM
cor = -0.524 p = 6.53e-31
partial.cor = -0.15 p = 2.15e-03
partial.cor = -0.292 p = 1.10e-09
partial.cor = 0.066 p = 1.79e-01
partial.cor = -0.092 p = 5.91e-02
partial.cor = 0.264 p = 4.42e-08
partial.cor = 0.562 p = 3.99e-36
HNSC
cor = -0.251 p = 1.61e-08
partial.cor = 0.076 p = 9.84e-02
partial.cor = 0.217 p = 1.80e-06
partial.cor = 0.186 p = 4.06e-05
partial.cor = 0.441 p = 2.27e-24
partial.cor = 0.206 p = 5.66e-06
partial.cor = 0.345 p = 7.00e-15
HNSC-HPVneg
cor = -0.192 p = 1.08e-04
partial.cor = 0.124 p = 1.41e-02
partial.cor = 0.301 p = 1.18e-09
partial.cor = 0.263 p = 1.17e-07
partial.cor = 0.472 p = 2.98e-23
partial.cor = 0.261 p = 1.66e-07
partial.cor = 0.402 p = 8.15e-17
HNSC-HPVpos
cor = -0.458 p = 5.58e-06
partial.cor = -0.086 p = 4.52e-01
partial.cor = -0.125 p = 2.74e-01
partial.cor = -0.112 p = 3.18e-01
partial.cor = 0.301 p = 4.91e-03
partial.cor = -0.027 p = 8.06e-01
partial.cor = 0.125 p = 2.58e-01
KICH
cor = -0.423 p = 4.07e-04
partial.cor = 0.237 p = 5.76e-02
partial.cor = -0.251 p = 4.38e-02
partial.cor = 0.146 p = 2.45e-01
partial.cor = -0.055 p = 6.62e-01
partial.cor = 0.014 p = 9.10e-01
partial.cor = 0.445 p = 2.03e-04
KIRC
cor = -0.176 p = 1.4e-04
partial.cor = 0.068 p = 1.49e-01
partial.cor = -0.056 p = 2.39e-01
partial.cor = -0.026 p = 5.78e-01
partial.cor = 0.205 p = 1.23e-05
partial.cor = 0.214 p = 3.90e-06
partial.cor = 0.109 p = 2.04e-02
KIRP
cor = -0.196 p = 1.5e-03
partial.cor = 0.354 p = 5.47e-09
partial.cor = 0.366 p = 1.29e-09
partial.cor = 0.277 p = 6.54e-06
partial.cor = 0.273 p = 1.20e-05
partial.cor = 0.366 p = 1.28e-09
partial.cor = 0.382 p = 2.49e-10

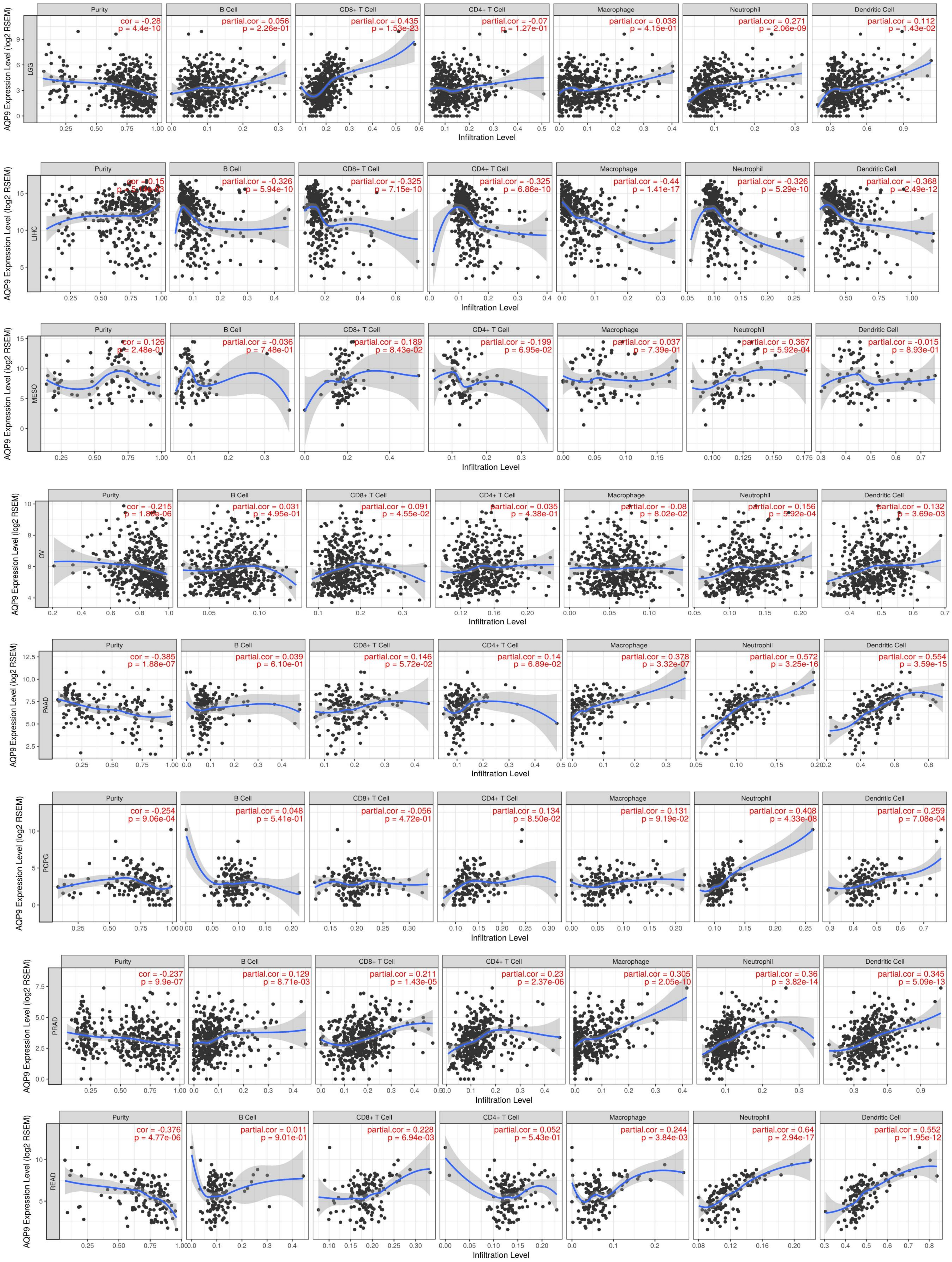
AQP9 Expression Level (log2 RSEM)
Infiltration Level
Purity
B Cell
CD8+ T Cell
CD4+ T Cell
Macrophage
Neutrophil
Dendritic Cell
LGG
cor = -0.28 p = 4.4e-10
partial.cor = 0.056 p = 2.26e-01
partial.cor = 0.435 p = 1.53e-23
partial.cor = -0.07 p = 1.27e-01
partial.cor = 0.038 p = 4.15e-01
partial.cor = 0.271 p = 2.06e-09
partial.cor = 0.112 p = 1.43e-02
LIHC
cor = 0.15
partial.cor = -0.326 p = 5.94e-10
partial.cor = -0.325 p = 7.15e-10
partial.cor = -0.325 p = 6.86e-10
partial.cor = -0.44 p = 1.41e-17
partial.cor = -0.326 p = 5.29e-10
partial.cor = -0.368 p = 2.49e-12
MESO
cor = 0.126 p = 2.48e-01
partial.cor = -0.036 p = 7.48e-01
partial.cor = 0.189 p = 8.43e-02
partial.cor = -0.199 p = 6.95e-02
partial.cor = 0.037 p = 7.39e-01
partial.cor = 0.367 p = 5.92e-04
partial.cor = -0.015 p = 8.93e-01
OV
cor = -0.215
partial.cor = 0.031 p = 4.95e-01
partial.cor = 0.091 p = 4.55e-02
partial.cor = 0.035 p = 4.38e-01
partial.cor = -0.08 p = 8.02e-02
partial.cor = 0.156 p = 5.92e-04
partial.cor = 0.132 p = 3.69e-03
PAAD
cor = -0.385 p = 1.88e-07
partial.cor = 0.039 p = 6.10e-01
partial.cor = 0.146 p = 5.72e-02
partial.cor = 0.14 p = 6.89e-02
partial.cor = 0.378 p = 3.32e-07
partial.cor = 0.572 p = 3.25e-16
partial.cor = 0.554 p = 3.59e-15
PCPG
cor = -0.254 p = 9.06e-04
partial.cor = 0.048 p = 5.41e-01
partial.cor = -0.056 p = 4.72e-01
partial.cor = 0.134 p = 8.50e-02
partial.cor = 0.131 p = 9.19e-02
partial.cor = 0.408 p = 4.33e-08
partial.cor = 0.259 p = 7.08e-04
PRAD
cor = -0.237 p = 9.9e-07
partial.cor = 0.129 p = 8.71e-03
partial.cor = 0.211 p = 1.43e-05
partial.cor = 0.23 p = 2.37e-06
partial.cor = 0.305 p = 2.05e-10
partial.cor = 0.36 p = 3.82e-14
partial.cor = 0.345 p = 5.09e-13
READ
cor = -0.376 p = 4.77e-06
partial.cor = 0.011 p = 9.01e-01
partial.cor = 0.228 p = 6.94e-03
partial.cor = 0.052 p = 5.43e-01
partial.cor = 0.244 p = 3.84e-03
partial.cor = 0.64 p = 2.94e-17
partial.cor = 0.552 p = 1.95e-12

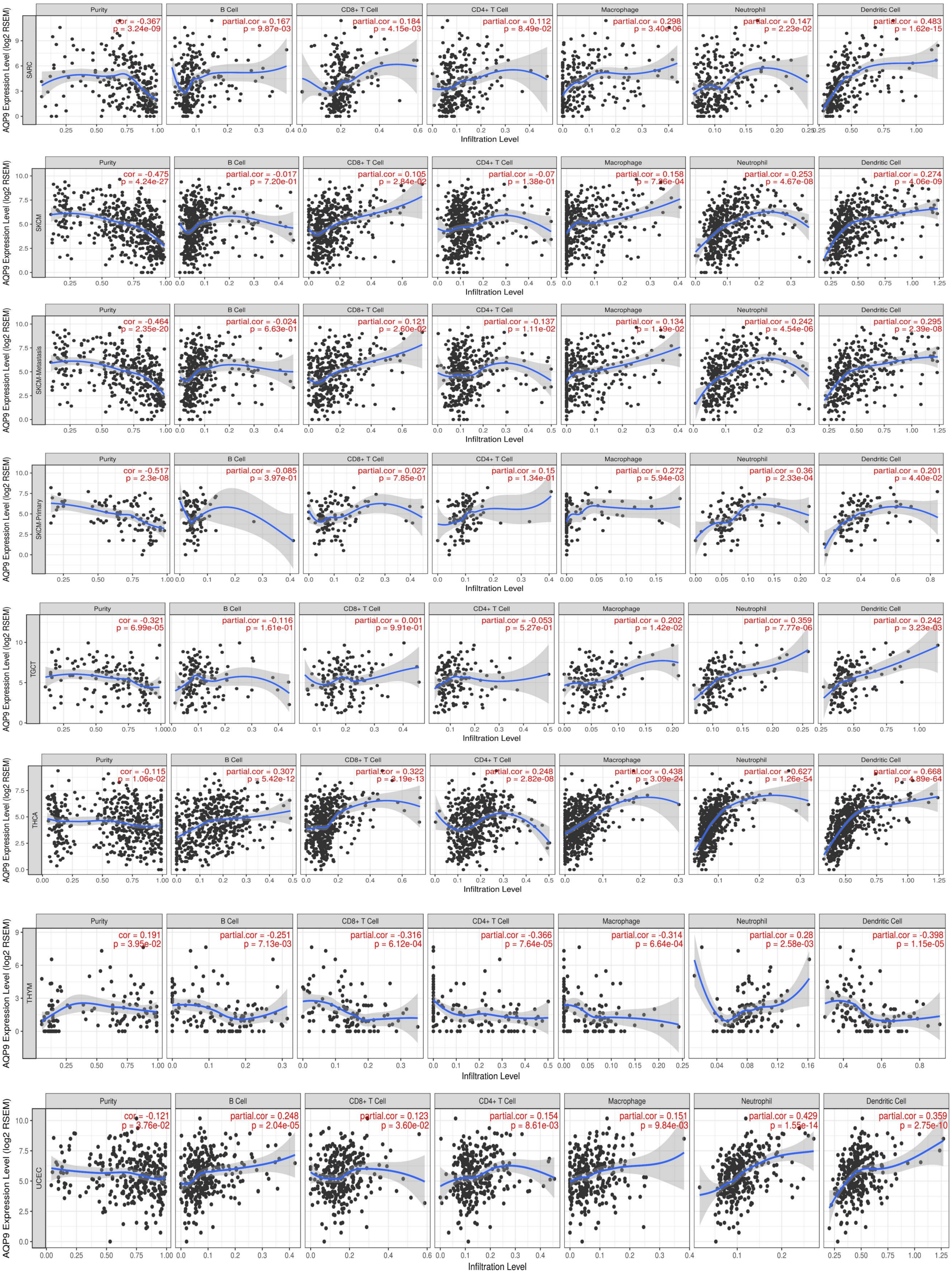

AQP9 Expression Level (log2 RSEM)
Infiltration Level
Purity
B Cell
CD8+ T Cell
CD4+ T Cell
Macrophage
Neutrophil
Dendritic Cell
SARC
cor = -0.367 p = 3.24e-09
partial.cor = 0.167 p = 9.87e-03
partial.cor = 0.184 p = 4.15e-03
partial.cor = 0.112 p = 8.49e-02
partial.cor = 0.298 p = 3.40e-06
partial.cor = 0.147 p = 2.23e-02
partial.cor = 0.483 p = 1.62e-15
SKCM
cor = -0.475 p = 4.24e-27
partial.cor = -0.017 p = 7.20e-01
partial.cor = 0.105 p = 2.84e-02
partial.cor = -0.07 p = 1.38e-01
partial.cor = 0.158 p = 7.86e-04
partial.cor = 0.253 p = 4.67e-08
partial.cor = 0.274 p = 4.06e-09
SKCM-Metastasis
cor = -0.464 p = 2.35e-20
partial.cor = -0.024 p = 6.63e-01
partial.cor = 0.121 p = 2.60e-02
partial.cor = -0.137 p = 1.11e-02
partial.cor = 0.134 p = 1.19e-02
partial.cor = 0.242 p = 4.54e-06
partial.cor = 0.295 p = 2.39e-08
SKCM-Primary
cor = -0.517 p = 2.3e-08
partial.cor = -0.085 p = 3.97e-01
partial.cor = 0.027 p = 7.85e-01
partial.cor = 0.15 p = 1.34e-01
partial.cor = 0.272 p = 5.94e-03
partial.cor = 0.36 p = 2.33e-04
partial.cor = 0.201 p = 4.40e-02
TGCT
cor = -0.321 p = 6.99e-05
partial.cor = -0.116 p = 1.61e-01
partial.cor = 0.001 p = 9.91e-01
partial.cor = -0.053 p = 5.27e-01
partial.cor = 0.202 p = 1.42e-02
partial.cor = 0.359 p = 7.77e-06
partial.cor = 0.242 p = 3.23e-03
THCA
cor = -0.115 p = 1.06e-02
partial.cor = 0.307 p = 5.42e-12
partial.cor = 0.322 p = 3.19e-13
partial.cor = 0.248 p = 2.82e-08
partial.cor = 0.438 p = 3.09e-24
partial.cor = 0.627 p = 1.26e-54
partial.cor = 0.668 p = 4.89e-64
THYM
cor = 0.191 p = 3.95e-02
partial.cor = -0.251 p = 7.13e-03
partial.cor = -0.316 p = 6.12e-04
partial.cor = -0.366 p = 7.64e-05
partial.cor = -0.314 p = 6.64e-04
partial.cor = 0.28 p = 2.58e-03
partial.cor = -0.398 p = 1.15e-05
UCEC
cor = -0.121 p = 3.76e-02
partial.cor = 0.248 p = 2.04e-05
partial.cor = 0.123 p = 3.60e-02
partial.cor = 0.154 p = 8.61e-03
partial.cor = 0.151 p = 9.84e-03
partial.cor = 0.429 p = 1.55e-14
partial.cor = 0.359 p = 2.75e-10

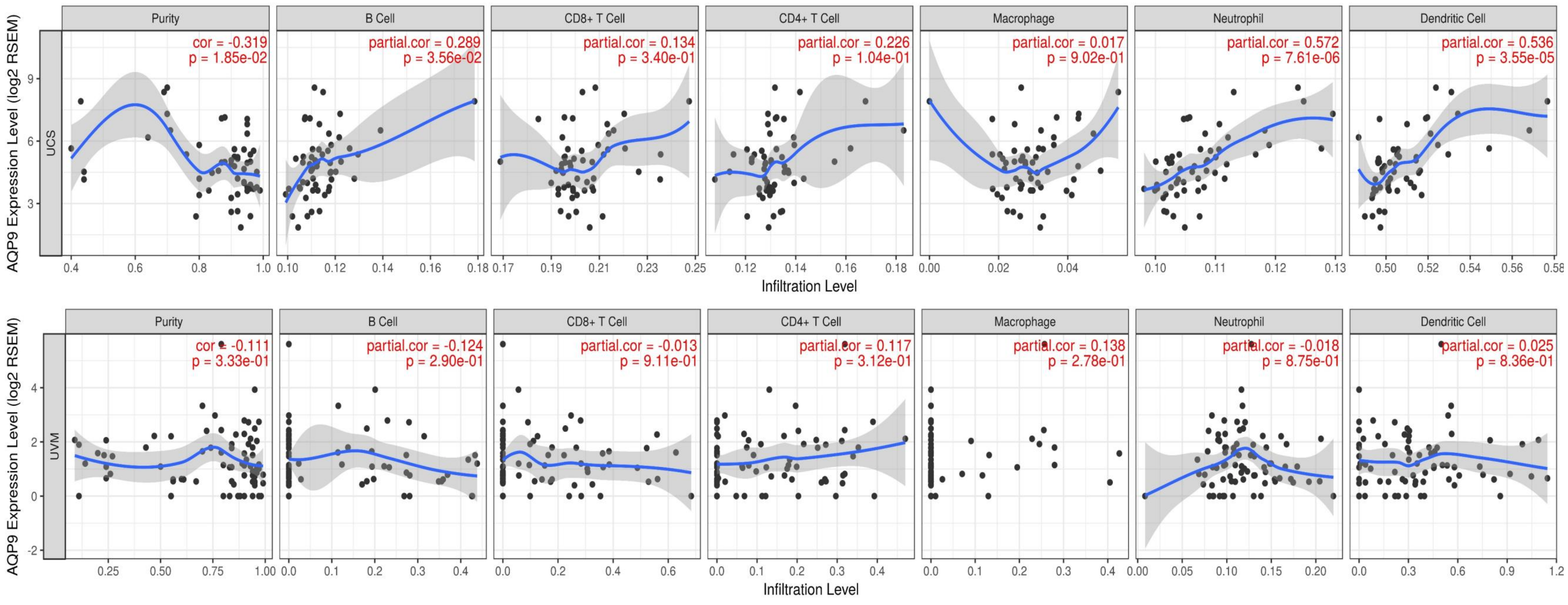

Supplementary Figure 5. Correlations of AQP9 expression with immune infiltrates levels in diverse types of cancer.

Supplementary Table 1. AQP9 expression in cancers vs normal tissue in oncomine database.

Cancer	Cancer type	*P*-value	Fold change	Rank (%)	Sample	Reference (PMID)
Bladder	Infiltrating Bladder Urothelial Carcinoma	6.09E-04	1.795	18%	129	16432078
	Superficial Bladder Cancer	2.69E-04	-1.907	17%	76	16432078
	Superficial Bladder Cancer	3.38E-04	-1.692	19%	14	15173019
Brain and CNS	Glioblastoma	8.07E-05	4.185	5%	25	16616334
	Oligodendroglioma	3.65E-06	-2.526	17%	73	16697959
Breast	Medullary Breast Carcinoma	4.24E-08	2.204	8%	176	22522925
	Ductal Breast Carcinoma	1.04E-04	2.186	10%	47	16473279
	Invasive Breast Carcinoma	1.15E-08	3.643	15%	137	TCGA
	Invasive Ductal Breast Carcinoma	1.91E-10	2.391	21%	450	TCGA
	Invasive Breast Carcinoma Stroma	3.91E-05	3.071	36%	59	18438415
Colorectal	Rectal Adenocarcinoma	6.33E-25	9.174	3%	130	20725992
	Colon Mucinous Adenocarcinoma	3.42E-08	8.761	8%	22	TCGA
	Colon Adenocarcinoma	2.00E-05	2.493	26%	22	TCGA
	Colorectal Carcinoma	1.65E-06	2.31	8%	60	20957034
	Cecum Adenocarcinoma	2.52E-04	1.702	14%	22	17615082
	Colorectal Carcinoma	3.94E-06	4.01	15%	82	20143136
	Colon Adenoma	1.10E-06	3.168	17%	57	18171984
	Colon Carcinoma	4.25E-04	2.005	23%	15	20957034
Esophageal	Esophageal Adenocarcinoma	2.28E-04	2.037	4%	33	16449976
	Esophageal Adenocarcinoma	2.03E-13	2.509	3%	103	21152079
Gastric	Gastric Intestinal Type Adenocarcinoma	1.26E-04	3.97	23%	57	19081245
Head and neck	Head and Neck Squamous Cell Carcinoma	2.48E-09	4.082	4%	54	14729608
	Oral Cavity Squamous Cell Carcinoma	8.05E-09	2.664	6%	79	21853135
Kidney	Non-Hereditary Clear Cell Renal Cell Carcinoma	6.57E-04	2.195	18%	11	19470766
Leukemia	Acute Myeloid Leukemia	2.08E-06	-69.221	1%	6	14770183
	B-Cell Acute Lymphoblastic Leukemia	7.54E-10	-14.271	6%	86	17410184
	Acute Myeloid Leukemia	3.00E-06	-5.519	8%	29	17410184
	T-Cell Acute Lymphoblastic Leukemia	2.84E-05	-15.332	12%	15	17410184
	Pro-B Acute Lymphoblastic Leukemia	7.28E-28	-4.391	5%	144	20406941
	Acute Myeloid Leukemia	5.06E-18	-2.65	5%	616	20406941
	B-Cell Acute Lymphoblastic Leukemia	4.63E-31	-4.712	5%	221	20406941
	B-Cell Childhood Acute Lymphoblastic Leukemia	1.60E-30	-4.608	6%	433	20406941
	T-Cell Acute Lymphoblastic Leukemia	2.60E-24	-3.565	7%	248	20406941
	Chronic Lymphocytic Leukemia	6.18E-23	-3.261	12%	522	20406941
Liver	Hepatocellular Carcinoma	1.20E-34	-4.48	5%	445	21159642
	Hepatocellular Carcinoma	5.41E-06	-8.787	6%	43	21159642
	Hepatocellular Carcinoma	6.45E-09	-2.753	9%	177	12058060
Lung	Lung Adenocarcinoma	7.91E-30	-3.559	1%	116	22613842
	Squamous Cell Lung Carcinoma	1.51E-05	-6.763	3%	38	11707567
	Lung Adenocarcinoma	1.77E-04	-4	8%	149	11707567
	Lung Carcinoid Tumor	3.11E-04	-3.868	27%	37	11707567
	Lung Adenocarcinoma	6.68E-05	-2.528	10%	57	17540040
	Large Cell Lung Carcinoma	7.65E-08	-5.065	9%	84	20421987
	Lung Adenocarcinoma	3.51E-08	-2.11	12%	110	20421987
	Squamous Cell Lung Carcinoma	2.03E-08	-3.079	14%	92	20421987
	Lung Adenocarcinoma	1.78E-07	-2.588	10%	246	22080568
Lymphoma	Diffuse Large B-Cell Lymphoma	9.75E-04	1.953	33%	64	19412164
Melanoma	Cutaneous Melanoma	1.34E-04	-6.182	3%	18	18442402
Ovarian	Ovarian Carcinoma	4.99E-08	-6.877	6%	195	18593951
Pancreatic	Pancreatic Carcinoma	6.28E-06	3.287	5%	52	19732725
	Pancreatic Ductal Adenocarcinoma	4.45E-04	1.815	32%	78	19260470
	Pancreatic Ductal Adenocarcinoma	7.96E-05	-1.588	2%	14	16103885
Prostate	Prostate Carcinoma Epithelia	4.04E-04	-2.973	4%	33	17173048
Sarcoma	Gastrointestinal Stromal Tumor	2.13E-05	-1.666	6%	25	21447720
	Leiomyosarcoma	2.56E-04	-2.151	12%	35	20601955
	Dedifferentiated Liposarcoma	5.89E-04	-1.952	13%	55	20601955
	Myxoid/Round Cell Liposarcoma	2.10E-04	-2.175	13%	29	20601955
Other	Embryonal Carcinoma, NOS	3.75E-07	1.977	6%	21	16424014
	Mixed Germ Cell Tumor, NOS	6.49E-06	1.529	17%	47	16424014
	Skin Basal Cell Carcinoma	9.27E-05	-3.757	2%	19	18442402

Supplementary Table 2. Correlation of AQP9 mRNA expression and clinical prognosis in breast cancer with different clinicopathological factors by Kaplan-Meier plotter.

Clinicopathological characteristic	Overall survival (n = 1402)			Recurrence-free survival (n = 3951)		
	N	Hazard ratio	P-value	N	Hazard ratio	P-value
**ER status**						
Positive	548	2.09(1.45-3.02)	**5.40E-05**	2061	1.62(1.37-1.91)	**7.00E-09**
Negative	251	1.04(0.66-1.64)	0.86	801	0.93(0.74-1.17)	0.54
**PR status**						
Positive	83	5.49(1.12-26.87)	**0.019**	589	1.86(1.3-2.66)	**0.00056**
Negative	89	1.37(0.54-3.48)	0.5	549	0.85(0.64-1.14)	0.27
**HER2 status**						
Positive	129	1.04(0.52-2.09)	0.91	252	0.78(0.51-1.21)	0.27
Negative	130	1.95(0.78-4.83)	0.14	800	1.63(1.25-2.13)	**0.00025**
**Intrinsic subtype**						
Basal	241	0.79(0.48-1.3)	0.35	618	0.93(0.72-1.19)	0.56
Luminal A	611	1.48(1.04-2.11)	**0.029**	1933	1.77(1.49-2.11)	**5.70E-11**
Luminal B	433	1.4(0.96-2.04)	0.079	1149	1.37(1.13-1.66)	**0.0013**
HER2+	117	0.97(0.51-1.86)	0.93	251	0.69(0.47-1.01)	0.057
**Lymph node status**						
Positive	313	1.24(0.84-1.83)	0.27	1133	1.51(1.24-1.84)	**3.60E-05**
Negative	594	1.93(1.32-2.81)	**0.00053**	2020	1.48(1.25-1.75)	**4.90E-05**
**Grade**						
1	161	2.15(0.84-5.49)	0.1	345	1.85(1.09-3.13)	**0.021**
2	387	1.51(0.98-2.32)	0.058	901	1.77(1.39-2.77)	**3.20E-06**
3	503	0.98(0.7-1.35)	0.88	903	1.03(0.83-1.28)	8.00E-01
**TP53 status**						
Mutated	111	1.36(0.63-2.92)	0.43	188	1.16(0.72-1.86)	0.54
Wild type	187	1.4(0.73-2.67)	0.31	273	1.39(0.91-2.12)	0.13
**Pietenpol subtype**						
Basal-like 1	58	0.86(0.25-2.99)	0.78	171	1.4(0.87-2.26)	0.17
Basal-like 2	39	3.33(0.85-13.08)	0.069	76	1.57(0.77-3.21)	0.21
Immunomodulatory	100	1.23(0.49-3.12)	0.66	203	1.11(0.61-2)	0.73
Mesenchymal	73	1.51(0.68-3.35)	0.31	177	1.23(0.0-1.88)	0.34
Luminal androgen receptor	83	1.07(0.54-2.11)	0.84	203	0.82(0.55-1.24)	0.35
**Systemic treatment**						
Without	382	1.71(1.09-2.69)	**0.018**	1010	1.51(1.22-1.87)	**0.00013**
With	1402	1.62(1.31-2.01)	**9.10E-06**	3951	1.61(1.45-1.8)	**<1E-16**

Bold values indicate P < 0.05.

Supplementary Table 3. Correlation of AQP9 mRNA expression and clinical prognosis in gastric cancer with different clinicopathological factors by Kaplan-Meier plotter.

Clinicopathological	Overall survival (n = 882)			Progression-free survival (n = 646)		
characteristic	N	Hazard ratio	P-value	N	Hazard ratio	P-value
**Sex**						
Female	236	0.55(0.38-0.8)	**0.0015**	201	0.64(0.43-0.96)	**0.028**
Male	545	0.69(0.55-0.87)	**0.0013**	438	0.68(0.58-0.89)	**0.0038**
**Stsge**						
1	67	0.15(0.03-0.67)	**0.0043**	60	0.2(0.04-0.9)	**0.019**
2	140	1.26(0.69-2.3)	0.44	131	1.32(0.69-2.56)	0.4
3	305	0.56(0.42-0.74)	**5.30E-05**	186	0.56(0.34-0.9)	**0.016**
4	148	1.33(0.88-2.02)	0.18	141	1.49(0.98-2.26)	0.063
**Stage T**						
2	241	0.66(0.41-1.07)	0.089	239	0.72(0.48-1.09)	0.12
3	204	0.72(0.51-1.02)	0.063	204	0.77(0.52-1.14)	0.19
4	38	0.38(0.14-1.01)	**0.043**	39	0.75(0.34-1.65)	0.47
**Stage N**						
0	74	0.26(0.11-0.62)	**0.0012**	72	0.26(0.11-0.62)	**0.0012**
1	225	0.85(0.56-1.28)	0.43	222	0.76(0.52-1.13)	0.17
2	121	0.55(0.35-0.86)	**0.0087**	125	0.58(0.33-1.01)	0.051
3	76	0.69(0.4-1.19)	0.18	76	0.81(0.47-1.39)	0.45
1+2+3	422	0.72(0.56-0.94)	**0.016**	423	0.77(0.59-0.99)	**0.04**
**Stage M**						
0	444	0.67(0.5-0.88)	**0.0046**	443	0.72(0.55-0.94)	**0.015**
1	56	0.69(0.37-1.29)	0.24	56	0.63(0.32-1.22)	0.17
**Lauren classification**						
Intestinal	320	0.69(0.48-0.99)	**0.04**	263	0.8(0.53-1.18)	0.26
Diffuse	241	0.58(0.41-0.83)	**0.0024**	231	0.6(0.42-0.86)	**0.0045**
**Differentiation**						
Poor	165	0.66(0.43-1.01)	0.055	121	0.73(0.46-1.16)	0.18
Moderate	67	1.36(0.69-2.69)	0.37	67	1.32(0.68-2.53)	0.41

Bold values indicate P < 0.05.

Supplementary Table 4. Correlation of AQP9 mRNA expression and clinical prognosis in lung cancer with different clinicopathological factors by Kaplan-Meier plotter.

Clinicopathological	Overall survival (n = 1928)			Progression-free survival (n = 982)		
characteristic	N	Hazard ratio	P-value	N	Hazard ratio	P-value
**Histology**						
Adenocarcinoma	720	2.06(1.63-2.61)	**6.90E-10**	461	1.84(1.33-2.54)	**0.00017**
Squamous cell carcinoma	524	1.39(1.07-1.82)	**0.014**	141	1.31(0.78-2.18)	0.31
**Stage**						
1	577	1.55(1.18-2.04)	**0.0016**	325	1.4(0.87-2.26)	0.16
2	244	1.41(0.97-2.06)	0.072	130	1.4(0.8-2.44)	0.24
3	70	2.66(1.37-5.19)	**0.0028**	19	-	-
**Grade**						
I	201	1.25(0.86-1.83)	0.24	140	1.36(0.79-2.35)	0.27
II	310	1.19(0.87-1.63)	0.28	165	1.48(0.96-2.26)	0.072
III	77	1.98(0.86-4.53)	0.1	51	1.59(0.7-3.58)	0.26
**Stage T**						
1	437	1.78(1.31-2.44)	**0.00022**	177	1.77(1.06-2.94)	**0.026**
2	589	1.11(0.89-1.39)	0.37	351	1.49(1.09-2.02)	**0.012**
3	81	1.63(0.93-2.87)	0.085	21	0.52(0.17-1.61)	0.25
4	46	1.76(0.82-3.77)	0.14	7	-	-
**Stage N**						
0	781	1.51(1.15-1.96)	**0.0023**	374	1.53(1.1-2.12)	**0.011**
1	252	1.58(1.14-2.19)	**0.006**	130	1.75(1.08-2.85)	**0.022**
2	111	1.31(0.83-2.08)	0.25	51	1.87(0.95-3.7)	0.066
**Stage M**						
0	681	1.34(1.08-1.66)	**0.0072**	195	1.59(0.95-2.67)	0.072
1	10	-	-	0	-	-
**Gender**						
Female	715	1.51(1.2-1.91)	**0.00047**	468	1.45(1.09-1.93)	**0.011**
Male	1100	1.27(1.08-1.49)	**0.0031**	514	1.78(1.36-2.34)	**2.50E-05**
**Smoking history**						
Exclude those never smoked	820	1.46(1.19-1.8)	**0.00027**	603	1.44(1.33-1.84)	**0.0031**
Only those never smoked	205	1.4(0.8-2.45)	0.23	193	1.38(0.86-2.22)	0.18

Bold values indicate P < 0.05.

Supplementary Table 5. Correlation analysis between AQP9 and relate genes and markers of immune cells in TIMER.

Description	Gene markers	BRCA				COAD				LUAD				LUSC				STAD			
		none		purity		none		purity		none		purity		none		purity		none		purity	
		cor	P	cor	P	cor	P	cor	P	cor	P	cor	P	cor	P	cor	P	cor	p	cor	P
CD8+ T cell	CD8A	0.188	***	0.161	***	0.430	***	0.349	***	0.207	***	0.090	*	0.419	***	0.327	***	0.070	0.156	0.031	0.543
	CD8B	0.188	***	0.164	***	0.242	***	0.211	***	0.184	***	0.096	*	0.335	***	0.284	***	-0.003	0.949	-0.027	0.599
T cell (general)	CD3D	0.245	***	0.218	***	0.357	***	0.256	***	0.210	***	0.083	0.067	0.482	***	0.358	***	0.113	*	0.060	0.241
	CD3E	0.241	***	0.214	***	0.401	***	0.295	***	0.188	***	0.045	0.319	0.493	***	0.362	***	0.062	0.207	0.001	0.984
	CD2	0.286	***	0.272	***	0.449	***	0.363	***	0.226	***	0.098	*	0.513	***	0.395	***	0.158	**	0.114	*
B cell	CD19	0.180	***	0.134	***	0.225	***	0.104	*	0.066	0.136	-0.077	0.086	0.338	***	0.154	***	-0.052	0.286	-0.088	0.089
	CD79A	0.161	***	0.118	***	0.267	***	0.128	**	0.101	*	-0.022	0.620	0.380	***	0.206	***	0.024	0.633	-0.021	0.678
Monocyte	CD86	0.522	***	0.527	***	0.817	***	0.788	***	0.656	***	0.620	***	0.725	***	0.647	***	0.542	***	0.524	***
	CD115 (CSF1R)	0.255	***	0.236	***	0.696	***	0.645	***	0.522	***	0.470	***	0.691	***	0.598	***	0.416	***	0.411	***
TAM	CCL2	0.449	***	0.439	***	0.720	***	0.690	***	0.495	***	0.457	***	0.562	***	0.493	***	0.311	***	0.266	***
	CD68	0.610	***	0.618	***	0.559	***	0.528	***	0.718	***	0.685	***	0.705	***	0.632	***	0.493	***	0.471	***
	IL10	0.546	***	0.555	***	0.673	***	0.654	***	0.527	***	0.463	***	0.618	***	0.555	***	0.486	***	0.463	***
M1 Macrophage	INOS (NOS2)	0.118	***	0.089	**	-0.003	0.951	-0.044	0.379	0.152	**	0.106	*	0.064	0.151	0.073	0.111	0.241	***	0.225	***
	IRF5	0.268	***	0.252	***	0.288	***	0.307	***	0.348	***	0.286	***	0.098	*	0.051	0.262	0.100	*	0.106	*
	COX2(PTGS2)	0.165	***	0.142	***	0.463	***	0.427	***	0.113	*	0.114	*	0.265	***	0.211	***	0.336	***	0.324	***
M2 Macrophage	CD163	0.620	***	0.629	***	0.827	***	0.802	***	0.713	***	0.680	***	0.782	***	0.729	***	0.590	***	0.581	***
	VSIG4	0.445	***	0.443	***	0.779	***	0.748	***	0.757	***	0.731	***	0.786	***	0.740	***	0.496	***	0.475	***
	MS4A4A	0.508	***	0.515	***	0.765	***	0.750	***	0.696	***	0.660	***	0.784	***	0.730	***	0.443	***	0.433	***
Neutrophils	CD66b (CEACAM8)	0.018	0.554	0.034	0.289	-0.020	0.664	0.040	0.424	0.140	**	0.129	**	0.196	***	0.181	***	0.108	*	0.114	*
	CD11b (ITGAM)	0.374	***	0.366	***	0.765	***	0.736	***	0.619	***	0.585	***	0.710	***	0.622	***	0.460	***	0.449	***
	CCR7	0.219	***	0.191	***	0.379	***	0.273	***	0.145	**	-0.004	0.933	0.421	***	0.273	***	0.095	0.054	0.053	0.301
	CD15(FUT4)	0.342	***	0.335	***	0.019	0.688	0.012	0.814	0.227	***	0.204	***	0.223	***	0.192	***	0.167	**	0.186	**
Natural killer cell	KIR2DL1	0.205	***	0.190	***	0.299	***	0.257	***	0.057	0.193	0.016	0.730	0.197	***	0.150	*	0.231	***	0.237	***
	KIR2DL3	0.195	***	0.177	***	0.280	***	0.219	***	0.240	***	0.180	***	0.244	***	0.199	***	0.223	***	0.229	***
	KIR2DL4	0.323	***	0.308	***	0.316	***	0.241	***	0.209	***	0.156	**	0.205	***	0.126	**	0.254	***	0.215	***
	KIR3DL1	0.241	***	0.222	***	0.344	***	0.276	***	0.175	***	0.130	**	0.303	***	0.240	***	0.121	*	0.105	*
	KIR3DL2	0.194	***	0.188	***	0.270	***	0.209	***	0.140	**	0.076	0.093	0.272	***	0.206	***	0.145	**	0.112	*
	KIR3DL3	0.073	*	0.068	*	0.143	**	0.122	*	0.133	**	0.123	**	0.118	**	0.105	*	0.147	**	0.154	**
	KIR2DS4	0.178	***	0.157	***	0.274	***	0.253	***	0.144	**	0.093	*	0.225	***	0.187	***	0.133	**	0.138	**
Dendritic cell	HLA-DPB1	0.155	***	0.118	***	0.595	***	0.532	***	0.292	***	0.205	***	0.625	***	0.527	***	0.202	***	0.158	**
	HLA-DQB1	0.197	***	0.170	***	0.420	***	0.347	***	0.178	***	0.095	*	0.520	***	0.417	***	0.197	***	0.152	**
	HLA-DRA	0.286	***	0.276	***	0.621	***	0.584	***	0.388	***	0.314	***	0.678	***	0.605	***	0.293	***	0.270	***
	HLA-DPA1	0.199	***	0.170	***	0.626	***	0.569	***	0.338	***	0.260	***	0.643	***	0.560	***	0.270	***	0.243	***
	BDCA-1(CD1C)	-0.035	0.242	-0.121	**	0.327	***	0.426	***	0.083	0.059	0.010	0.827	0.392	***	0.195	***	-0.031	0.533	-0.079	0.125
	BDCA-4(NRP1)	0.145	***	0.118	**	0.723	***	0.684	***	0.212	***	0.199	***	0.503	***	0.405	***	0.286	***	0.267	***
	CD11c (ITGAX)	0.502	***	0.504	***	0.825	***	0.795	***	0.554	***	0.498	***	0.716	***	0.630	***	0.592	***	0.578	***
Th1	T-bet (TBX21)	0.268	***	0.251	***	0.445	***	0.383	***	0.191	***	0.066	0.141	0.416	***	0.284	***	0.149	**	0.113	*
	STAT4	0.240	***	0.218	***	0.485	***	0.415	***	0.217	***	0.113	*	0.525	***	0.401	***	0.163	***	0.132	*
	STAT1	0.398	***	0.395	***	0.516	***	0.478	***	0.281	***	0.207	***	0.281	***	0.209	***	0.161	**	0.141	**
	IFN-γ (IFNG)	0.322	***	0.312	***	0.384	***	0.359	***	0.237	***	0.146	**	0.263	***	0.196	***	0.248	***	0.225	***
	TNF-α (TNF)	0.297	***	0.280	***	0.511	***	0.501	***	0.305	***	0.221	***	0.389	***	0.268	***	0.359	***	0.342	***
Th2	STAT5A	0.010	0.733	-0.026	0.414	0.203	***	0.183	**	0.330	***	0.239	***	0.474	***	0.344	***	0.135	**	0.122	*
	IL13	0.214	***	0.194	***	0.336	***	0.271	***	-0.015	0.740	0.053	0.229	0.228	***	0.151	***	0.093	0.057	0.090	0.079
Tfh	BCL6	0.002	0.955	-0.011	0.728	0.513	***	0.433	***	-0.036	0.420	-0.046	0.303	-0.127	**	-0.105	*	0.042	0.393	0.003	0.952
	IL21	0.204	***	0.193	***	0.310	***	0.303	***	0.181	***	0.122	**	0.236	***	0.139	**	0.282	***	0.263	***
Th17	STAT3	0.018	0.544	0.027	0.389	0.318	***	0.266	***	0.013	0.767	0.018	0.686	0.219	***	0.152	***	0.193	***	0.179	***
	IL17A	0.172	***	0.174	***	-0.081	0.084	-0.099	*	0.098	0.262	0.037	0.409	0.109	*	0.038	0.411	0.139	**	0.118	*
Treg	FOXP3	0.383	***	0.368	***	0.493	***	0.423	***	0.250	***	0.149	**	0.476	***	0.333	***	0.220	***	0.193	***
	CCR8	0.424	***	0.419	***	0.538	***	0.478	***	0.334	***	0.260	***	0.548	***	0.441	***	0.334	***	0.322	***
	STAT5B	-0.188	***	-0.202	***	0.078	0.096	0.091	0.068	0.080	0.068	0.061	0.176	0.012	0.795	-0.011	0.804	-0.017	0.734	-0.005	0.922
	TGFβ (TGFB1)	0.004	0.891	0.053	0.078	0.591	***	0.516	***	0.242	***	0.180	***	0.216	***	0.083	0.069	0.138	**	0.115	*
T cell exhaustion	PD-1 (PDCD1)	0.268	***	0.246	***	0.389	***	0.307	***	0.194	***	0.076	0.093	0.373	***	0.238	***	0.120	*	0.091	0.076
	CTLA4	0.437	***	0.435	***	0.501	***	0.443	***	0.268	***	0.151	**	0.469	***	0.332	***	0.221	***	0.186	***
	LAG3	0.395	***	0.380	***	0.456	***	0.368	***	0.161	**	0.065	0.148	0.295	***	0.185	***	0.219	***	0.179	***
	TIM-3 (HAVCR2)	0.552	***	0.561	***	0.801	***	0.772	***	0.671	***	0.634	***	0.741	***	0.681	***	0.533	***	0.515	***
	GZMB	0.452	***	0.451	***	0.232	***	0.227	***	0.366	***	0.291	***	0.421	***	0.313	***	0.343	***	0.306	***

BRCA, breast invasive carcinoma; COAD, colon adenocarcinoma; LUAD, lung adenocarcinoma; LUSC, lung squamous cell carcinoma; STAD, stomach adenocarcinoma; TAM, tumor-associated macrophage; Th, T helper cell; Tfh, follicular helper T cell; Treg, regulatory T cell; Cor, R value of Spearman's correlation; None, correlation without adjustment. Purity, correlation adjusted by purity. *P < 0.05; **P < 0.01; ***P < 0.001.

Supplementary Table 6. Correlation analysis between AQP9 and relate genes and markers of monocyte and macrophages in GEPIA.

Gene markers	BRCA				COAD				LUAD				LUSC				STAD			
	Tumor		Normal		Tumor		Normal		Tumor		Normal		Tumor		Normal		Tumor		Normal	
	R	P	R	P	R	P	R	P	R	P	R	P	R	P	R	P	R	P	R	P
CD86	0.5	***	0.74	***	0.83	***	0.079	0.62	0.66	***	0.65	***	0.72	***	0.54	***	0.59	***	0.73	***
CD115 (CSF1R)	0.25	***	0.69	***	0.78	**	0.049	0.76	0.54	***	0.5	***	0.68	***	0.44	**	0.47	***	0.6	***
CCL2	0.43	***	0.42	***	0.74	***	0.3	0.058	0.49	***	0.26	*	0.56	***	0.21	0.14	0.35	***	0.3	0.08
CD68	0.59	***	0.81	***	0.69	***	0.16	0.33	0.72	***	0.34	**	0.68	***	0.53	***	0.57	***	0.3	0.08
IL10	0.5	***	0.7	***	0.7	***	0.31	0.051	0.52	***	0.42	***	0.58	***	0.27	0.06	0.52	***	0.69	***
INOS (NOS2)	0.12	***	-0.27	**	0.067	0.27	-0.087	0.59	0.21	***	-0.18	0.17	0.075	0.10	-0.11	0.43	0.25	***	0.38	*
IRF5	0.24	***	0.51	***	0.39	***	0.15	0.35	0.35	***	0.41	**	0.082	0.07	0.37	**	0.2	***	0.046	0.79
COX2(PTGS2)	0.17	***	0.017	0.86	0.5	***	0.51	***	0.14	**	0.11	0.40	0.29	***	0.13	0.36	0.38	***	0.3	0.07
CD163	0.63	***	0.77	***	0.84	***	0.19	0.24	0.7	***	0.41	**	0.76	***	0.45	**	0.53	***	0.17	0.33
VSIG4	0.43	***	0.74	***	0.81	***	0.16	0.33	0.76	***	0.48	***	0.77	***	0.64	***	0.53	***	0.43	**
MS4A4A	0.5	***	0.73	***	0.81	***	0.31	*	0.71	***	0.51	***	0.77	***	0.61	***	0.51	***	0.42	*

BRCA, breast invasive carcinoma; COAD, colon adenocarcinoma; LUAD, lung adenocarcinoma; LUSC, lung squamous cell carcinoma; S TAD, stomach adenocarcinoma; TAM, tumor-associated macrophages. Tumor, correlation analysis in tumor tiss ue of TCGA. Normal, correlation analysis in normal tissue of TCGA. *P < 0.0 5; **P < 0.01; ***P < 0.001.
